# Analysis of Benefit of Intensive Care Unit Transfer for Deteriorating Ward Patients: A Patient-Centered Approach to Clinical Evaluation

**DOI:** 10.1001/jamanetworkopen.2018.7704

**Published:** 2019-02-15

**Authors:** Richard Grieve, Stephen O’Neill, Anirban Basu, Luke Keele, Kathryn M. Rowan, Steve Harris

**Affiliations:** 1 Department of Health Services Research and Policy, London School of Hygiene and Tropical Medicine, London, United Kingdom; 2 J.E. Cairnes School of Business & Economics, National University of Ireland, Galway, Ireland; 3 The Comparative Health Outcomes, Policy, and Economics (CHOICE) Institute, University of Washington, Seattle; 4 Center for Surgery and Health Economics, University of Pennsylvania, Philadelphia; 5 Intensive Care National Audit & Research Centre (ICNARC), London, United Kingdom; 6 Division of Medicine, University College London, London, United Kingdom

## Abstract

**Importance:**

It is unknown which deteriorating ward patients benefit from intensive care unit (ICU) transfer.

**Objectives:**

To use an instrumental variable (IV) method that assesses heterogeneity and to evaluate estimates of person-centered treatment effects of ICU transfer and 28-day hospital mortality by age and illness severity.

**Design, Setting, and Participants:**

An analysis of a prospective cohort study from November 1, 2010, to December 31, 2011. The dates of this analysis were June 1, 2017, to June 30, 2018. The setting was a multicenter study of 49 UK National Health Service hospitals. Participants were 9192 deteriorating ward patients assessed for ICU transfer (4596 matched pairs). The study matched on baseline characteristics to strengthen the IV and to balance observed confounders between the comparison groups.

**Exposures:**

Transfer to the ICU or continued care on general wards.

**Main Outcomes and Measures:**

Mortality at 28 days (primary outcome) and 90 days. To address unobserved confounding, ICU bed availability was the IV for whether or not a patient was transferred. The study used the IV approach to evaluate estimates of treatment effect of ICU transfer and mortality according to age and physiological severity alone and in combination.

**Results:**

Both comparison groups included 4596 patients. In the group assessed with “many” ICU beds available (median, 7), 52.8% were male, and the mean (SD) age was 65.2 (17.7) years; in the group assessed with “few” ICU beds available (median, 2), 53.3% were male, and the mean (SD) age was 65.0 (17.3) years. The overall 28-day mortality estimates were 23.2% (2090 predicted deaths) if all of the matched patients were transferred vs 28.1% (2534 predicted deaths) if none of the matched patients were transferred, an estimated risk difference of −4.9% (95% CI, −26.4% to 16.6%). The estimated effects of ICU transfer differed by age and by physiological severity according to the National Early Warning Score (NEWS): the absolute risk differences in 28-day mortality after ICU transfer ranged from 7.7% (95% CI, −5.5% to 21.0%) for ages 18 to 23 years to −5.0% (95% CI -26.5% to 16.6%) for age 78 to 83 years and ranged from 3.7% (95% CI, −12.1% to 19.5%) for NEWS of 0 to −25.4% (95% CI, −50.6% to −0.2%) for NEWS of 19. The absolute risk differences for elderly patients (≥75 years) were −11.6% (95% CI, −39.0% to 15.8%) for those with high NEWS (>6), −4.8% (95% CI, −30.5% to 20.9%) for those with moderate NEWS (5-6), and −1.0% (95% CI, −24.8% to 22.8%) for those with low NEWS (<5). The corresponding estimates for subgroups of younger patients (<75 years) were −8.4% (95% CI, −31.0% to 14.1%), −2.1% (95% CI, −21.1% to 16.9%), and 1.4% (95% CI, −14.5% to 17.4%).

**Conclusions and Relevance:**

This study using a this person-centered IV approach found that the benefits of ICU care may increase among patients at high levels of baseline physiological severity across different age groups, especially among elderly patients.

## Key Points

**Question:**

Which deteriorating ward patients benefit from intensive care unit transfer?

**Findings:**

This analysis of a cohort study of 4596 deteriorating ward patients used an instrumental variable approach to evaluate estimates of person-centered effects of ICU transfer and mortality. This study found an increased risk reduction in 28-day mortality in patients transferred to ICU who were older than 75 years and had greater illness severity (National Early Warning Scores >6).

**Meaning:**

The instrumental variable approach in this study found that benefits of intensive care unit transfer may increase with age and baseline physiology score.

## Teaser

This cohort study uses an instrumental variable method to assess heterogeneity and reports estimated person-centered treatment effects of intensive care unit (ICU) transfer with 28-day hospital mortality by age and illness severity among deteriorating ward patients.

## Introduction

The proportion of intensive care unit (ICU) patients who are elderly is increasing.^1^ Intensive care unit costs are 1% of the gross domestic product in the United States and are potentially unsustainable.^2,3^ Previous observational studies^4,5,6^ reported that ICU transfer was associated with increased mortality but relied on risk adjustment methods that did not address confounding by indication or recognize potential heterogeneity in the effectiveness of ICU care according to the patient’s prognosis. There are major practical and ethical challenges in undertaking randomized clinical trials to evaluate ICU transfer for deteriorating ward patients. A recent cluster randomized clinical trial^7^ reported that a program to increase ICU admissions for elderly patients did not reduce hospital mortality. These findings may discourage ICU transfer for older patients^8,9,10^ or reinforce treatment limitations, despite concerns that the conclusions of the randomized clinical trial^7^ did not apply to routine clinical practice.^2^

Precision or personalized medicine aims to provide the right interventions for the right patients at the right times, which can be initiated according to any measures of the individual patient’s prognosis and is not restricted to his or her genetic or biomarker profile.^11,12,13^ In particular, the gains from ICU transfer may depend on prognostic factors, such as age, which may modify the effectiveness of ICU transfer alone, and in synergy with measures, such as the patient’s physiological status.^14,15^ A major challenge in generating evidence to support clinical decision making is that the gains from ICU care may differ according to unmeasured characteristics, such as frailty, which may also determine whether or not the patient is transferred to the ICU. Previous studies^14,15^ have not recognized that the effectiveness of ICU care may differ according to unmeasured patient-level factors.

A new method for fully examining heterogeneity that addresses confounding by indication reports person-centered treatment (PeT) effects.^16,17,18^ This method uses insights about how clinicians select interventions according to the anticipated health gain for each patient to provide individual-level estimates of treatment effectiveness, which are then aggregated to subgroups of prime interest.^16,17,18,19^ We revisit a natural experiment that used ICU bed availability as an instrumental variable (IV) for ICU transfer to estimate the effectiveness of transfer for a subsample of deteriorating ward patients.^20^ In contrast to the previous study, which focused on transfer within 4 hours, we estimate the effectiveness of ICU transfer per se for all patients assessed. We fully assess heterogeneity in the effectiveness of ICU transfer according to observed and unobserved characteristics across all eligible deteriorating ward patients. To our knowledge, this article is the first to recognize that the effectiveness of ICU care may be modified according to patient characteristics that are unmeasured and those that are measured.^17,20,21,22^ We estimate the effectiveness of ICU transfer according to patient age and illness severity alone and combined.

## Methods

### Data Source, Participants, and Definitions

This prospective cohort study was conducted from November 1, 2010, to December 31, 2011. The dates of this analysis were June 1, 2017, to June 30, 2018. We used data from the (SPOT)light study,^20^ a prospective cohort of deteriorating ward patients assessed for possible transfer to the ICU in 49 UK National Health Service hospitals. This study followed the Strengthening the Reporting of Observational Studies in Epidemiology (STROBE) reporting guideline.^20,23^ We excluded repeat visits, readmissions, deaths during the assessment, admissions after surgery (when admission may be protocolized), and patients with preexisting treatment limitations. Intensive care unit admission and survival were defined by linkage to the Intensive Care National Audit & Research Centre (ICNARC) Case Mix Programme database and the National Health Service Information Service, respectively. The study was registered on the National Institute of Health Research research portfolio (No. 9139) and with ClinicalTrials.gov (NCT0101099813). Ethical approval was provided by the National Health Service National Research Ethics Committee (Cambridgeshire 3) on September 2, 2010, for study protocol version 1.1. Consent was not obtained, but permission to process patient data was approved by the National Information Governance Board Ethics & Confidentiality Committee. The full study protocol is available on the ICNARC website.^24^

The treatment variable was transfer from a ward to the ICU, and the primary outcome was mortality up to 28 days after ICU assessment. The IV was ICU bed availability, defined as the sum of physically empty ICU beds plus those with ICU patients ready for discharge.^20,21,25,26^ This measure has been previously used as an IV for patient admission or transfer.^20,21,25,26^ Following these studies, we assumed that spare ICU capacity encouraged transfer but did not otherwise affect the mortality of patients assessed.

Baseline covariates were all recorded by the bedside clinician at assessment for transfer and included age, diagnosis of sepsis, periarrest (a clinical judgment of impending cardiorespiratory arrest), and the physiological measures required to calculate the following 3 acute physiological scores: the National Health Service National Early Warning Score (NEWS),^27^ the ICNARC physiology score,^28^ and the Sequential Organ Failure Assessment (SOFA).^29^ The patient’s existing dependency at ward assessment and recommended level of care after assessment were defined by UK Critical Care Minimum Data Set levels of care, equivalent to general ward care (levels 0 and 1), high-dependency care (level 2), and ICU (level 3).^30^ The data included indicators of low ICU capacity, including whether ward assessment was during the weekend (Saturday or Sunday), outside of regular hours (7 pm to 7 am), or outside of the winter months (April through November).

### Statistical Analysis

#### Near-Far Matching

We paired patients with similar baseline characteristics and matched patients assessed when there were “many” vs “few” ICU beds available, excluding matched pairs with a difference of less than 3 ICU beds available (eAppendix 1 in the Supplement).^31^ This near-far matching algorithm attempted to ensure that measured confounders were balanced and the instrument (ICU bed availability) was strong (ie, predicted ICU transfer).^32,33,34,35^ To assess the quality of the matches, we reported covariate balance according to standardized mean differences.

We assessed whether the IV design met the essential criteria for validity. First, we calculated the partial *F* statistic to indicate IV strength^36^ and found that the number of available beds was highly correlated with ICU transfer (*F* statistic, 71). Second, we found that the measured baseline risk factors were balanced according to the number of available ICU beds (eFigure 1 in the Supplement).

#### Approach to Estimating PeT Effects Using the IV Design

We estimated average PeT effects to fully account for heterogeneity and confounding (eAppendices 2, 3, and 4 in the Supplement).^17,18,19,37,38^ This IV approach recognizes that the transfer decision reflects unmeasured and measured patient and contextual factors. We identified “marginal” patients for whom the physician was in equipoise about the transfer decision according to their measured (eg, ICU beds available and physiological score) and unmeasured (eg, frailty) characteristics (eFigure 2 in the Supplement). For these patients, a small change (or nudge) in the number of ICU beds available (the IV) “tips the balance” for the decision to transfer the patient to the ICU but does not change the distribution of his or her risk factors. Comparing outcomes for patients defined according to small differences in the ICU beds available provides an estimate of the effect of ICU transfer for similar patients. By repeating this contrast across different numbers of beds available (the IV), we estimate treatment effects for sets of marginal patients with different combinations of confounders (eg, age and NEWS). For each individual, a PeT effect is obtained by averaging the treatment effects for those marginal patients who share the same observed and unobserved characteristics according to their observed treatment choice. The PeT effects can be averaged over any sample characteristics to form a subgroup-level mean effect.

**Figure 1.**
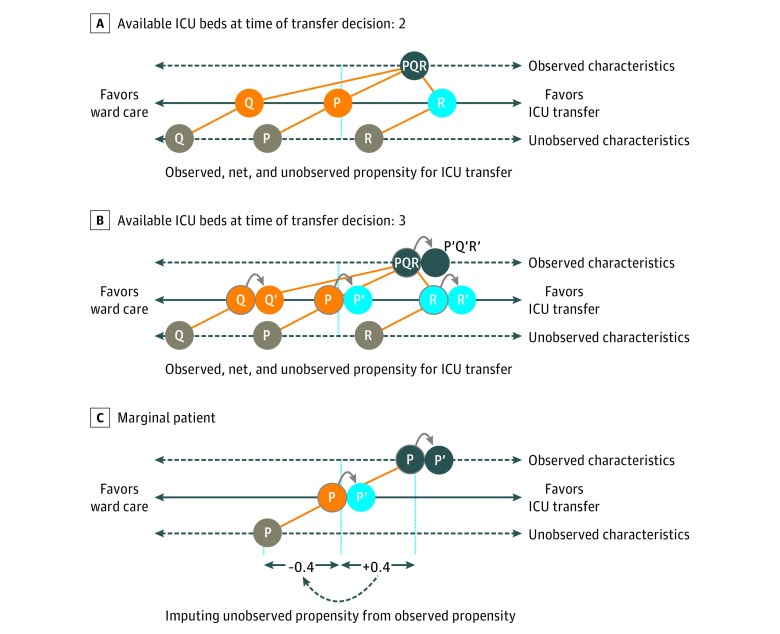
Essence of the Person-Centered Treatment Approach A patient’s propensity for transfer is gauged according to observed and unobserved characteristics. Three patients (P, Q, and R) share the same propensity according to observed factors. However, the propensity for intensive care unit (ICU) transfer according to unobserved characteristics is low for patient Q, balanced for patient P, and high for patient R. An additional bed only affects the decision to transfer for P, the “marginal” patient. A, Patient P is marginal in that the propensity to transfer according to observed and unobserved characteristics is balanced. With 2 beds available, the marginal patient remains on the general ward. B, The additional bed availability (3 beds) encourages the decision to transfer the marginal patient (P→P′). C, For the marginal patient, we proxy the propensity for ICU transfer according to unobserved characteristics as the negative of the propensity due to observed characteristics. For this marginal patient, the treatment effect is estimated by accounting for observed and unobserved characteristics.

For illustration, Figure 1 shows 3 patients (P, Q, and R) assessed for ICU transfer. In making the transfer decision, the physician considers each patient’s expected outcome, acknowledging the ICU bed capacity. Only some of the information the physician uses to make this decision is measured and available for the study (eg, age and NEWS); other confounders (eg, frailty) are unavailable. Here, patient Q has a low propensity for transfer according to unobserved characteristics and is not transferred, whereas patient R has a high overall propensity for transfer and is transferred. With just 2 beds available (Figure 1A), patient P is defined as marginal in that the physician is indifferent about transferring the patient, who remains on the general ward.

With 3 ICU beds available (Figure 1B), the physician is nudged to recommend transfer for patient P′. We use this information about the decision to transfer to infer that, for patients P and P′, the observed and unobserved confounders must exactly balance. We use this insight to derive the distributions of unobservable characteristics and contrast outcomes between sets of marginal patients who are nudged toward (like patient P′) or away (like patient P) from ICU transfer by the number of available beds. By contrasting outcomes for alternative sets of marginal patients according to different levels of bed availability, we estimate PeT effects for each patient while accounting for unobserved confounding and heterogeneity. We average these individual PeT effects for each patient across observed confounders of interest to derive minimally biased estimates of heterogeneity in the effectiveness of ICU transfer.

The person-level IV approach was implemented as follows. First, we estimated each patient’s propensity for ICU transfer according to his or her observed characteristics and the number of available ICU beds. Second, we used these estimates of propensity for ICU transfer vs the observed transfer status to estimate each marginal patient’s “residual” propensity for transfer according to his or her unobserved characteristics (Figure 1C). Third, we used this estimated propensity for transfer according to unobserved factors to allow for unobserved confounding when predicting outcomes after ICU transfer vs general ward care (eAppendix 5 in the Supplement).

The PeT effect was defined for each patient in the matched data as the difference in his or her predicted 28-day mortality with vs without ICU transfer. These person-level estimated treatment effects were aggregated to report the stimated effects of ICU transfer over the whole matched sample and for each prespecified subgroup of interest (age, NEWS, ICNARC physiology score, and SOFA score) and for each physiology measure combined with age. The effectiveness of ICU transfer was reported for patients 75 years and older or younger than 75 years^21^ for low (<5), moderate (5 or 6), or high (>6) levels of baseline risk according to NEWS^27^ and for the secondary end point of 90-day mortality. Because both the exposure and the outcomes were binary, probit regression models were used to estimate the PeT effects. The effectiveness of ICU transfer was reported as the mean (95% CI) differences in the absolute risk of death.

Finally, we developed a simple predictive model (logistic regression) to examine which baseline characteristics predicted the magnitude of the estimated absolute risk difference in 28-day mortality (eAppendix 6 in the Supplement). We defined the magnitude of clinical benefit as a 10% difference in 28-day mortality.^20^ All standard errors were calculated with nonparametric bootstrapping and allowed for clustering of individuals within hospitals, with inferences conditional on the matched data. Data analyses were performed in R (version 3.4.3; R Foundation for Statistical Computing) and in Stata (version 14.1; StataCorp LP).

### Sensitivity Analyses

To test whether the findings were robust to alternative choices, we considered alternative statistical models, including different functional forms (probit vs logit) for the treatment and outcome equations, the inclusion vs exclusion of higher-order terms for continuous baseline measures, and interaction terms between each physiology score. We contrasted these estimates with a traditional IV approach, 2-stage least squares, which reports estimated effectiveness for a subpopulation of deteriorating ward patients,^22^ and to a regression approach that assumes no unmeasured confounders (eAppendix 7 in the Supplement).

## Results

A total of 13 011 eligible patients were assessed for ICU transfer, of whom 4994 (38.4%) were transferred. Before matching, the ICU-transferred patients were, on average, younger, more often had a diagnosis of sepsis, and were more severely ill (higher NEWS, ICNARC physiology score, and SOFA score) (eTable 1 in the Supplement).

Among deteriorating ward patients assessed for ICU transfer, the near-far matching algorithm identified 4596 matched pairs. In the matched sample (N = 9192), 52.8% of the many (median, 7) ICU beds group were male, and the mean (SD) age was 65.2 (17.7) years; 53.3% of the few (median, 2) ICU beds group were male, and the mean (SD) age was 65.0 (17.3) years. Other baseline characteristics were also well balanced between the comparison groups (Table 1) and similar for the matched vs unmatched samples (eTable 2 in the Supplement).

**Table 1.** Baseline Characteristics After Matching for Admissions With Many vs Few Intensive Care Unit (ICU) Beds Available at Time of Assessment for ICU Transfer

Variable	Many ICU Beds	Few ICU Beds	Standardized Difference
No. of admissions	4596	4596	NA
No. of ICU beds available			
Mean (SD)	7.64 (2.67)	1.68 (1.13)	2.905
Median (range)	7 (5-19)	2 (0-3)	NA
Transfer to ICU, No. (%)	1995 (43.4)	1521 (33.1)	0.213
Age, mean (SD), y	65.23 (17.68)	65.00 (17.35)	0.013
Male, No. (%)	2426 (52.8)	2448 (53.3)	−0.010
Reported sepsis diagnosis, No. (%)	2868 (62.4)	2873 (62.5)	−0.002
CCMDS level of care at visit, No. (%)			
0	496 (10.8)	604 (13.1)	−0.072
1	3247 (70.6)	3162 (68.8)	0.040
2	755 (16.4)	792 (17.2)	−0.022
3	54 (1.2)	30 (0.7)	0.055
Missing	44 (1.0)	8 (0.2)	0.105
Periarrest, No. (%)	233 (5.1)	164 (3.6)	0.074
Acute physiological scores, mean (SD)			
NEWS^a^	6.18 (3.12)	6.28 (3.05)	−0.030
ICNARC physiology score^b^	15.07 (7.40)	15.23 (7.12)	−0.021
SOFA score^c^	3.14 (2.19)	3.16 (2.15)	−0.011
NEWS risk class, No. (%)			
None	117 (2.5)	128 (2.8)	−0.015
Low	1244 (27.1)	1150 (25.0)	0.047
Medium	1287 (28.0)	1304 (28.4)	−0.008
High	1948 (42.4)	2014 (43.8)	−0.029
Time of admission, No. (%)			
Weekend	1172 (25.5)	1059 (23.0)	0.057
Outside of regular hours	1549 (33.7)	1649 (35.9)	−0.046
Winter	960 (20.9)	960 (20.9)	−0.000

Abbreviations: CCMDS, Critical Care Minimum Data Set; ICNARC, Intensive Care National Audit & Research Centre; NA, not applicable; NEWS, National Early Warning Score; SOFA, Sequential Organ Failure Assessment.

^a^ The NEWS ranges from 0 (least severe) to 20 (most severe).

^b^ The ICNARC physiology score ranges from 0 (least severe) to 100 (most severe).

^c^ The SOFA score ranges from 0 (least severe) to 14 (most severe).

The overall 28-day mortality estimates were 23.2% (2090 predicted deaths) if all of the matched patients were transferred vs 28.1% (2534 predicted deaths) if none of the matched patients were transferred, an estimated risk difference of −4.9% (95% CI, −26.4% to 16.6%) (Table 2). The 2-stage least squares approach also showed a lower 28-day mortality after ICU transfer (eTable 3 in the Supplement), whereas the regression and unadjusted approaches, which did not allow for unobserved confounding, demonstrated that ICU transfer was associated with an increase in 28-day mortality (Table 2).

**Table 2.** Overall 28-Day Mortality After Intensive Care Unit (ICU) vs General Ward Care for the Matched Sample

Estimator	Sample Size^a^	ICU Deaths, No. (%)^b^	General Ward Deaths, No. (%)^b^	Risk Difference, % (95% CI)^c^
IV (PeT, Probit)	9015	2090 (23.2)	2534 (28.1)	−4.9 (−26.4 to 16.6)
IV (PeT, Logit)	9015	2096 (23.2)	2539 (28.2)	−4.9 (−24.4 to 16.6)
Regression	9192	2594 (28.2)	1914 (20.8)	7.4 (5.0 to 9.8)
Unadjusted	9192	2915 (31.7)	1715 (18.7)	13.1 (11.2 to 14.9)

Abbreviations: IV, instrumental variable; PeT, person-centered treatment.

^a^ For each method, the maximum sample size was 9192. Observations were excluded if there is not mass at any value (rounded to 0.01) of the propensity score for both levels of exposure as recommended by Basu.^18^

^b^ The number of predicted deaths is rounded to the nearest whole number.

^c^ Normal-based 95% CI with standard error is calculated with the nonparametric bootstrap, allowing for clustering by hospital. Difference in percentage of deaths is from the PeT instrumental variable analysis.

**Figure 2.**
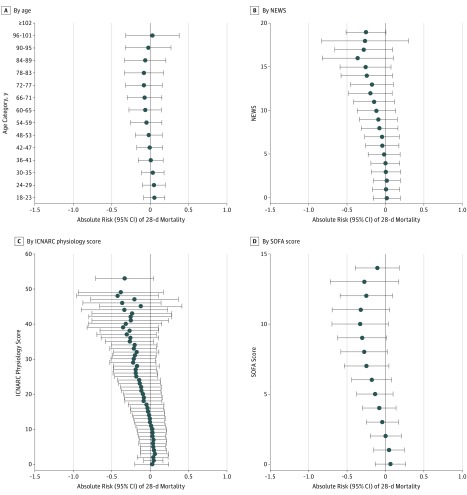
Estimated Person-Centered Treatment Effects of Intensive Care Unit Transfer vs General Ward Care With 28-Day Mortality Person-centered treatment effects by strata. Absolute risk reductions (95% CIs) are shown. Heterogeneous effects are estimated for each individual using the person-centered treatment method and then aggregated according to strata. The National Health Service National Early Warning Score (NEWS) ranges from 0 (least severe) to 20 (most severe). The Intensive Care National Audit & Research Centre (ICNARC) physiology score ranges from 0 (least severe) to 100 (most severe). The Sequential Organ Failure Assessment (SOFA) score ranges from 0 (least severe) to 14 (most severe).

The PeT estimated risk differences according to physiological severity varied from 3.7% (95% CI, −12.1% to 19.5%) for NEWS of 0 to −25.4% (95% CI, −50.6% to −0.2%) for NEWS of 19, with a similar pattern observed according to baseline ICNARC physiology score or SOFA score (Figure 2 and eTable 4 in the Supplement). The estimated risk differences by age varied from 7.7% (95% CI, −5.5% to 21.0%) for age 18 to 23 years to −5.0% (95% CI, -26.5% to 16.6%) for ages 72 to 77 years (Figure 2 and eTable 4 in the Supplement). The predicted risk difference after ICU transfer was −10.1% (95% CI, −33.2% to 13.0%) for those who were actually transferred and 3.3% (95% CI, −15.2% to 21.8%) for those who were not (eTable 4 and eFigure 3 in the Supplement).

The absolute risk differences in 28-day mortality after ICU transfer for elderly patients (≥75 years) were −11.6% (95% CI, −39.0% to 15.8%) for those with high NEWS (>6), −4.8% (95% CI, −30.5% to 20.9%) for those with moderate NEWS (5-6), and −1.0% (95% CI, −24.8% to 22.8%) for those with low NEWS (<5). The estimates for corresponding subgroups of younger patients (<75 years) were −8.4% (95% CI, −31.0% to 14.1%), −2.1% (95% CI, −21.1% to 16.9%), and 1.4% (95% CI, −14.5% to 17.4%) (eTable 5 in the Supplement).

**Figure 3.**
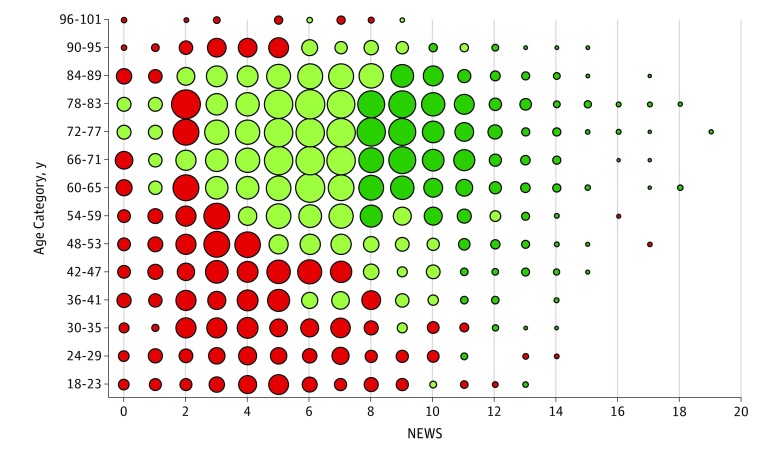
Bubble Chart Showing Estimated Person-Centered Treatment Effects of Intensive Care Unit Transfer vs General Ward Care on 28-Day Mortality, by Age Category and NEWS A National Health Service National Early Warning Score (NEWS) of less than 5 is considered low risk of 28-day mortality, 5 to 6 as moderate, and greater than 6 as high. Dark green indicates levels of absolute risk reductions exceeding 10%, light green as 0% to 10% risk reduction, and red as increased absolute risk of 28-day mortality. A larger dot indicates more individuals in that subgroup.

Figure 3 shows that the patients predicted to have reductions of greater than 10% in the absolute risk of 28-day mortality were those with high NEWS (>7) at assessment, who were also 60 years and older. Those patients predicted to have risk reductions between 0% and 10% had NEWS of 3 to 7 and were 50 years and older. The groups predicted to have higher 28-day mortality after ICU transfer were younger patients (<60 years), who had lower NEWS (<4) at assessment (eFigure 4 in the Supplement).

Baseline characteristics associated with absolute risk reductions in 28-day mortality after ICU transfer of greater than 10% were assessed. These characteristics included age; male sex; higher levels of care at assessment; periarrest; higher NEWS, ICNARC physiology score, and SOFA score; and admissions during core working hours (Monday through Friday, 9 am to 5 pm) and not in winter (eTable 6 and eTable 7 in the Supplement).

The overall PeT results for 90-day mortality were similar (eTable 8 in the Supplement), and the same pattern of heterogeneity was present; the patients predicted to benefit most from ICU transfer were older and more severely ill at assessment (eTable 9 and eFigure 5 in the Supplement). The supplementary analyses demonstrated that the results were robust to alternative ways of implementing the PeT approach (eTable 10 and eTable 11 in the Supplement). The findings were similar with the baseline ICNARC physiology score or SOFA score (eFigure 4 and eFigure 5 in the Supplement), as well as if those patients who were transferred later (after 24 hours) or recommended for ICU (level 3) care were excluded (eTable 12, eTable 13, eFigure 6, and eFigure 7 in the Supplement).

## Discussion

This person-centered IV approach addressed heterogeneity and confounding according to unobserved factors and found that the benefits of ICU transfer increase with age and physiology score. Patients with low physiology scores at assessment did not appear to benefit from ICU transfer. The study findings help identify which patients benefit most from ICU transfer and can inform future triage policies that aim to deliver additional clinical benefits within current ICU capacity. These findings bring into question recommendations that rely on estimates of the average effectiveness of ICU transfer; these estimates may either encourage unsustainable expansion of critical care^20,21^ or restrict access for elderly patients irrespective of their physiological status.^7^

Our patient-centered approach suggests that age and physiological severity act in synergy to predict the likely benefits and harms from ICU care for individual patients. The finding that the benefits from ICU transfer were greater in those recommended for transfer suggests that physicians were already personalizing their approach and triaging according to the likely gain for individual patients (eFigure 3 in the Supplement). Our finding that ICU transfer was beneficial for patients with higher physiological score at assessment extends previous observational studies^20,21^ of the effectiveness of ICU transfer for patients that used a traditional IV design. The findings from these previous studies only apply to a subsample of patients, whereas our person-level IV approach provided overall estimates of the effect of ICU care that apply to patients assessed for ICU care more generally.

One potential concern for the generalizability of the results is that, in the United Kingdom, the low levels of bed availability may imply that the patients considered for admission are more severely ill than in other countries. There are few international comparisons of severity of illness, but a patient-level meta-analysis^39^ of early goal-directed therapy (PRISM investigators) reported that the median Acute Physiology and Chronic Health Evaluation II physiology score at randomization was only slightly higher in the United Kingdom (12; interquartile range, 8-17) compared with the United States (10; interquartile range, 7-15). Moreover, we present estimates of the effectiveness of ICU care disaggregated according to commonly used measures of case severity to help inform clinical decision making in different contexts.

### Strengths and Limitations

The study has several strengths. First, this IV approach originally developed in the econometrics literature^17,18^ was able to identify which types of patients benefited most from ICU transfer. Second, the article extended the previously published study^20^ that used the same IV design by addressing interpatient heterogeneity. Third, the study reports that the main findings are robust to a battery of alternative assumptions, notably those underlying the IV approach.

This study has potential limitations. First, IV analyses are notoriously statistically inefficient,^40^ but the IV approach described herein demonstrated smaller standard errors around the absolute risk differences compared with traditional IV methods. Second, any subgroup analysis raises a concern about spurious claims of statistical significance. Therefore, we prespecified hypotheses about which risk factors were anticipated to modify the effect of ICU transfer with mortality^21^ and exploited the rich set of baseline measures available from the ICNARC Case Mix Programme database. Nevertheless, further research to test the replication of these findings would be useful.^41^ Third, to assist future clinical decision makers, we developed a risk prediction algorithm. While the algorithm has face validity according to the variables that predicted mortality gains from ICU transfer of greater than 10%, it warrants testing in other contexts before it could be considered for use in routine clinical practice.

## Conclusions

To our knowledge, this is the first study to evaluate ICU transfer with an IV method that simultaneously recognizes patient heterogeneity and addresses confounding by indication. The gains from ICU transfer were greater for older patients and for patients at higher levels of physiological severity at assessment. This approach can help improve clinical outcomes from limited ICU capacity by informing decisions about triage for ICU care and can inform moves to personalize clinical decision making more widely.

## Notes

Supplement. **eAppendix 1.** Near-far matching, prior to instrumental variable (IV) estimation **eAppendix 2.** The intuitive ideas behind essential heterogeneity, marginal treatment effects and person-centered treatment (PeT) effects **eAppendix 3.** Formal models behind essential heterogeneity, marginal treatment effects and person-centered treatment (PeT) effects **eAppendix 4.** Estimating MTE and PeT effects **eAppendix 5.** Implementation of PeT for evaluating the effectiveness of ICU transfer for deteriorating ward patients **eAppendix 6.** Details of predictive models **eAppendix 7.** Sensitivity Analysis **eReferences.** **eTable 1.** Baseline characteristics of all deteriorating ward patients receiving ICU versus General Ward care, before matching. **eTable 2.** Baseline characteristics for the patients in the unmatched versus matched samples **eTable 3.** Comparison of overall effects, estimated by PeT versus 2-stage least squares (2LS). **eTable 4.** Summary of effects on 28-day mortality, by subgroup. **eTable 5.** Summary of effects on 28- and 90-day mortality, by age group and level of NEWS score. **eTable 6.** Baseline characteristics explaining variations in PeT effects following ICU transfer versus general ward care on 28-day mortality. **eTable 7.** Local instrumental variable (LIV) model for 28-day mortality. **eTable 8.** 90-day Mortality following ICU versus General Ward care for the matched sample. **eTable 9.** Summary of effects of ICU transfer on 90-day mortality, by subgroup. **eTable 10.** Sensitivity analysis of conditional average treatment effects on 28-day mortality by subgroup. **eTable 11.** Sensitivity analysis of conditional average treatment effects on 90-day mortality, overall and by subgroup **eTable 12.** 28- and 90-day Mortality following ICU versus General Ward care for the matched sample excluding patients transferred after 24 hours **eTable 13.** 28- and 90-day Mortality following ICU versus General Ward care for the matched sample excluding patients recommended for ICU (level 3) **eFigure 1.** Mean level of rescaled variables according to the level of the instrumental variable. **eFigure 2.** Illustration of plausible values for unobserved covariates influencing transfer decision **eFigure 3.** Estimated PeT effects of ICU transfer versus general ward care on 28-day mortality, overall and by subgroup according to whether ICU transfer was recommended, and whether the patient was actually transferred **eFigure 4.** Bubble chart indicating average effect of being transferred to ICU by other subgroups. **eFigure 5.** Estimated PeT effects of ICU transfer versus general ward care on 90-day mortality, according to strata, defined by age and physiology scores at assessment for ICU transfer. **eFigure 6.** Sensitivity analysis, excluding patients transferred to ICU more than 24 hours after admission. Bubble chart indicating estimated PeT effects of ICU transfer versus general ward care on 28-day mortality, by age category and NEWS score. **eFigure 7.** Sensitivity analysis, excluding patients recommended for level 3 (ICU) care post assessment. Bubble chart indicating estimated PeT effects of ICU transfer versus general ward care on 28-day mortality, by age category and NEWS score. **eFigure 8.** Distribution of potential effects of transfer to ICU on 28- and 90-day mortality according to actual admission.
