# Supplementary material for: Analysis of Benefit of Intensive Care Unit Transfer for Deteriorating Ward Patients: A Patient-Centered Approach to Clinical Evaluation
Source: JAMA Netw Open. 2019 Feb 15;2(2):e187704. doi: 10.1001/jamanetworkopen.2018.7704 (PMC6484590; doi:10.1001/jamanetworkopen.2018.7704)
Supplement: Supplement. — eAppendix 1. Near-far matching, prior to instrumental variable (IV) estimation eAppendix 2. The intuitive ideas behind essential heterogeneity, marginal treatment effects and person-centered treatment (PeT) effects eAppendix 3. Formal models behind essential heterogeneity, marginal treatment effects and person-centered treatment (PeT) effects eAppendix 4. Estimating MTE and PeT effects eAppendix 5. Implementation of PeT for evaluating the effectiveness of ICU transfer for deteriorating ward patients eAppendix 6. Details of predictive models eAppendix 7. Sensitivity Analysis eReferences. eTable 1. Baseline characteristics of all deteriorating ward patients receiving ICU versus General Ward care, before matching. eTable 2. Baseline characteristics for the patients in the unmatched versus matched samples eTable 3. Comparison of overall effects, estimated by PeT versus 2-stage least squares (2LS). eTable 4. Summary of effects on 28-day mortality, by subgroup. eTable 5. Summary of effects on 28- and 90-day mortality, by age group and level of NEWS score. eTable 6. Baseline characteristics explaining variations in PeT effects following ICU transfer versus general ward care on 28-day mortality. eTable 7. Local instrumental variable (LIV) model for 28-day mortality. eTable 8. 90-day Mortality following ICU versus General Ward care for the matched sample. eTable 9. Summary of effects of ICU transfer on 90-day mortality, by subgroup. eTable 10. Sensitivity analysis of conditional average treatment effects on 28-day mortality by subgroup. eTable 11. Sensitivity analysis of conditional average treatment effects on 90-day mortality, overall and by subgroup eTable 12. 28- and 90-day Mortality following ICU versus General Ward care for the matched sample excluding patients transferred after 24 hours eTable 13. 28- and 90-day Mortality following ICU versus General Ward care for the matched sample excluding patients recommended for ICU (level 3) eFigure 1. Mean level of rescaled var [file jamanetwopen-2-e187704-s001.pdf]

Supplementary Online Content

Grieve R, O'Neill S, Basu A, Keele L, Rowan KM, Harris S. Analysis of benefit of intensive care unit transfer to deteriorating ward patients: a patient-centered approach to clinical evaluation. *JAMA Netw Open*. 2019;2(2):e187704.
doi:10.1001/jamanetworkopen.2018.7704

eAppendix 1. Near-far matching, prior to instrumental variable (IV) estimation

eAppendix 2. The intuitive ideas behind essential heterogeneity, marginal treatment effects and person-centered treatment (PeT) effects

eAppendix 3. Formal models behind essential heterogeneity, marginal treatment effects and person-centered treatment (PeT) effects

eAppendix 4. Estimating MTE and PeT effects

eAppendix 5. Implementation of PeT for evaluating the effectiveness of ICU transfer for deteriorating ward patients

eAppendix 6. Details of predictive models

eAppendix 7. Sensitivity analysis

eReferences.

eTable 1. Baseline characteristics of all deteriorating ward patients receiving ICU versus General Ward care, before matching.

eTable 2. Baseline characteristics for the patients in the unmatched versus matched samples

eTable 3. Comparison of overall effects, estimated by PeT versus 2-stage least squares (2LS).

eTable 4. Summary of effects on 28-day mortality, by subgroup.

eTable 5. Summary of effects on 28- and 90-day mortality, by age group and level of NEWS score.

eTable 6. Baseline characteristics explaining variations in PeT effects following ICU transfer versus general ward care on 28-day mortality.

eTable 7. Local instrumental variable (LIV) model for 28-day mortality.

eTable 8. 90-day Mortality following ICU versus General Ward care for the matched sample

eTable 9. Summary of effects of ICU transfer on 90-day mortality, by subgroup.

eTable 10. Sensitivity analysis of conditional average treatment effects on 28-day mortality by subgroup.

eTable 11. Sensitivity analysis of conditional average treatment effects on 90-day mortality, overall and by subgroup

eTable 12. 28- and 90-day Mortality following ICU versus General Ward care for the matched sample excluding patients transferred after 24 hours

eTable 13. 28- and 90-day Mortality following ICU versus General Ward care for the matched sample excluding patients recommended for ICU (level 3)

© 2019 Grieve R et al. *JAMA Network Open*.

eFigure 1. Mean level of rescaled variables according to the level of the instrumental variable.

eFigure 2. Illustration of plausible values for unobserved covariates influencing transfer decision

eFigure 3. Estimated PeT effects of ICU transfer versus general ward care on 28-day mortality, overall and by subgroup according to whether ICU transfer was recommended, and whether the patient was actually transferred

eFigure 4. Bubble chart indicating average effect of being transferred to ICU by other subgroups.

eFigure 5. Estimated PeT effects of ICU transfer versus general ward care on 90-day mortality, according to strata, defined by age and physiology scores at assessment for ICU transfer.

eFigure 6. Sensitivity analysis, excluding patients transferred to ICU more than 24 hours after admission. Bubble chart indicating estimated PeT effects of ICU transfer versus general ward care on 28-day mortality, by age category and NEWS score.

eFigure 7. Sensitivity analysis, excluding patients recommended for level 3 (ICU) care post assessment. Bubble chart indicating estimated PeT effects of ICU transfer versus general ward care on 28-day mortality, by age category and NEWS score.

eFigure 8. Distribution of potential effects of transfer to ICU on 28- and 90-day mortality according to actual admission.

This supplementary material has been provided by the authors to give readers additional information about their work.

eAppendix 1: Near-Far matching, prior to Instrumental Variable (IV) estimation.

1.1 Overview

Near-far matching is a matched-pair IV study design that aims to reduce weak instrument bias by ensuring that within the matched pairs, units are 'far' apart on the instrument but 'near' according to other baseline covariates [1-3]. Hence there are *I* matched pairs, with the units in each pair indexed by *j* ={1,2}, which are 'near' according to observed covariates (**x**), i.e. $\mathbf{x}_{i1} \approx \mathbf{x}_{i2}$, but far according to the instrument (**Z**), implying ($\mathbf{Z}_{i1}$ - $\mathbf{Z}_{i2}$) is large for each matched pair *i*=1,…*I***.**

1.2 Application of the near-far matching algorithm to strengthen the IV in the (SPOT)light study, while balancing baseline prognostic variables

We use the same matched data as a previous paper that assessed the overall effectiveness of ICU transfer using the (SPOT)light data. For full details of the matching algorithm, interested readers are referred to the paper by Keele et al (2019)[4], and so here we provide an overview of the key features.

The aim of the near-far matching algorithm was to strengthen the instrument, the number of beds available at the time of assessment, while balancing the baseline covariates between the groups under comparison. The comparison groups are defined as those patients assessed when there were 'many' versus 'few' ICU beds available. To obtain the matched dataset, Keele et al (2019) first calculated the pairwise distance between patients included in the sample using the rank-based Mahalanobis distance metric which is robust to low-incidence binary variables and variables with highly skewed distributions [5]. For two covariates, the current level of care, and the recommended level of care, a small fraction of data were missing. Instead of imputing these missing values based on a model, a method recommended by Rosenbaum (2010) [5] was used. Missing values were imputed using the mean for that covariate and a separate indicator for whether the value was missing was created. The imputed values were included in the distance calculation. The indicators for missing data were subsequently included in the match to ensure that the rate of missingness was balanced across comparison groups.

Next, to obtain the near-far match, Keele et al (2019) matched with a reverse caliper[1] so that only those matches where the difference in the instrumental variable (Z_{i1} - Z_{i2}) exceeded a threshold, Λ,

---

[1] While typically caliper matching attempts to avoid poor matches by imposing a tolerance on the maximum distance between matched pairs (Cochran and Rubin 1973), Keele et al use a caliper to reject matches by imposing a tolerance on the *minimum* distance between matched pairs.

© 2019 Grieve R et al. *JAMA Network Open*.

were acceptable. The larger the required difference in the instrumental variable, the more difficult it may be to find those units similar according to observed covariates. Hence, to balance covariates while strengthening the instrument, it may be necessary to remove some observations. Here, the matching algorithm used optimal subset matching [6], which seeks to find the largest set of matched pairs such that the average matched distance within a pair did not exceed a particular threshold $\tilde{\delta}$. The parameter $\tilde{\delta}$ can also be viewed as a penalty, describing the cost of excluding a treated individual from the match. In general, as the value for $\tilde{\delta}$ is increased, sufficiently large samples meet the average distance criterion, so that the match does not exclude anyone (at the cost of greater covariate imbalance), and as $\tilde{\delta}$ is decreased, more units will be excluded so that only the closest pairs will be retained in the match.

Keele et al (2019) undertook a grid search which iterated over values of Λ and $\tilde{\delta}$ to produce those matches judged to lead to sufficient balance, and instrument strength. In this study, Λ=1.5 times the standard deviation of the instrumental variable (number of beds available) and $\tilde{\delta}$ =1000 for the average matched distance, were judged most appropriate. Choosing Λ=1.5, resulted in the exclusion of those matched pairs where the difference between the matched patients was that there were less than three beds available in the ICU at the time of assessment. Hence, patients were excluded from the matched data if it was not possible to find a corresponding patient with similar covariates but a difference of at least three ICU beds available at assessment. The net result was that the sample size was reduced from 13,011 (unmatched) to 9,192 patients (4,596 matched pairs), but the characteristics of the patients included in the matching were similar to those excluded (eTable 2).

1.3 Performance of the matching algorithm

Following the near-far matching, the balance of the matched data according to the level of the instrument was assessed for each baseline covariate. First, the standardized mean differences were reported across two groups defined as the subsamples of patients who were admitted when there were 'many' versus 'few' beds available. Here the results show that the standardized differences were relatively low (all less than 10) (see main text, Table 1). Second, the levels of the covariates (rescaled by their standard deviations) were compared according to the levels of the IV, the number of beds available. If the covariates influencing outcomes tend not to vary across levels of the IV (as is the case here (eFigure 1), this increases confidence that the IV only influences outcomes through its influence on the likelihood of ICU transfer. Overall, the near-far matching algorithm provides good covariate balance across the levels of the IV.

© 2019 Grieve R et al. *JAMA Network Open*.

As intended, the near-far matching algorithm also increased the strength of the instrument, assessed using the Cragg-Donald Wald F-statistic for weak instruments and by comparing the proportion of patients transferred to ICU when there were 'many' versus 'few' beds available. In the near-far matched data, the proportion of patients transferred to ICU was 10.3 percentage points higher when 'many' beds were available (43.4%) than when 'few' beds were available (33.1%) and the Cragg-Donald Wald F-statistic for weak instruments was 70.962. For comparison, when the instrument is not strengthened, that is when a 'near' matching algorithm was used instead, the difference in the proportion of patients transferred to ICU was only 7.1 percentage points (33.9% versus 41%) and the Cragg-Donald Wald F-statistic was 63.247.

According to these results the near-far matching algorithm balanced the observed covariates, that is: age, gender, NEWS SOFA and ICNARC physiology scores, CCMDS level at assessment, and timing (out of hours, winter, and weekend or not) while increasing the imbalance for the IV as desired (STROBE checklist).

We now describe the intuition behind the particular IV approach taken to report the effectiveness of ICU transfer for deteriorating ward patients.

eAppendix 2: The intuitive ideas behind essential heterogeneity, marginal treatment effects (MTEs) and person-centered treatment (PeT) effects.

2.1 Essential heterogeneity

Studies examining the impact of ICU care often distil comparisons down to a single number that represents the average incremental benefit or harm [7,8]. This approach ignores evidence that there is substantial variability in the case-mix of patients admitted to ICU, and the effectiveness of ICU care may be heterogeneous. A further challenge is that the selection of patients for ICU transfer is according to risk factors that modify the effectiveness of ICU care, and many of these factors, such as the patient's frailty or pre-admission health status may remain unmeasured. In particular, clinicians may select those patients for ICU transfer according to their anticipated gain in health outcome, but their health status at assessment is not measured in the data recorded.

These issues limit the usefulness of traditional approaches that report the effectiveness of ICU care overall, or for a limited range of measured patient subgroups. Together, heterogeneous effects of ICU transfer and selection into ICU based on anticipated gains are termed 'essential heterogeneity'. In the presence of this 'essential heterogeneity' previous methodological research has shown that

© 2019 Grieve R et al. *JAMA Network Open*.

traditional IV regressions tend to estimate a local average treatment effect parameter that is often not interpretable, or of clinical relevance. In particular, the resultant estimate only applies to the ill-defined subgroup who would have switched treatment modality according to a change in the level of the instrument [8-15].

We address these concerns with a recently developed econometric methodology that uses an instrumental variable (IV) to address selection biases in observational studies and establish person-centered treatment (PeT) effects [16-18]. PeT effects estimate an average treatment effect for each person in the data, conditioning on their observed characteristics and the level of the IV, and crucially accounting for their individualized distribution of unobserved heterogeneity (see next section). Consequently, such individualized effects can help answer distributional questions on effectiveness, such as examining the benefits and harms of ICU care versus care in a general ward, and identifying subgroups that are most likely to benefit from such care.

2.2 Marginal treatment effects (MTEs)

To provide the intuition behind these concepts, we pose a clinical question; is transfer to ICU effective for deteriorating ward patients? Some clinicians believe that the effectiveness of ICU care differs according to patients underlying health condition and age, and select patients for ICU accordingly. One challenge for the analysis is that not all of the required variables are measured. Suppose the available dataset contains for each patient: age, mortality, and a valid instrumental variable, the number of ICU beds available at assessment (NBA). The fewer the NBA, the less likely the patient is to be transferred to ICU.

A local instrumental variable (LIV) approach can be used to overcome the problem of essential heterogeneity when a multivalued instrument, such as NBA, is available. LIV methods are used to estimate the marginal treatment effects (MTEs) parameters. MTEs are the treatment effects for those individuals for whom the influence of the observed characteristics (age and NBA in the stylized example), balance with those of the unobserved confounders (medical history) on the decision to transfer the patient, such that the clinician is indifferent to the decision as to whether or not to transfer the patient to ICU (see Figure 1 in main text).

To estimate an MTE, the LIV approach compares the outcomes of two groups of similar patients (say aged 50), where one group is faced with a constraint of *d* beds available and the other a slightly weaker constraint d+ε, with ε representing a slight increase in beds available. These two groups of patients should be identical with respect to the distribution of their risk factors (observed and

© 2019 Grieve R et al. *JAMA Network Open*.

unobserved) provided NBA is independent of all risk factors affecting outcomes. Hence the individual's propensity for transfer is identical, beyond the slight difference in the constraint according to the number of beds. By definition, this independence assumption will hold if NBA is a valid instrumental variable (assumption 2 below). The decision as to whether to transfer these similar patients to ICU is only according to the NBA, which does not directly influence outcomes (assumption 2 below). Therefore any difference in average outcomes between these two groups is only driven by the receipt of ICU care or not, for this margin of patients where the clinicians were indifferent between transfer and not, but were nudged to transfer by the small perturbation of the instrumental variable, i.e. NBA.

For this margin of patients, we can quantify a normalized level of unobserved confounders that was sufficient to balance their observed confounders at the considered level of NBA (*d*). Here, normalized means a scalar score that represents a balancing score for unobserved risk factors, irrespective of their empirical distributions. One can think of the normalized level of unobserved confounders as the propensity not to transfer the patient based on unobserved confounders. If the observed and unobserved risk factors do not balance, then the small perturbation induced by the nudge would have been inadequate to affect treatment selection. The obvious but crucial consequence is that we can use this insight to quantify the effect of the unmeasured confounders on treatment selection.

By definition for marginal patients, the propensity to transfer the patient to ICU equals the propensity not to. The difference in average outcomes between the two groups of similar patients (e.g. aged 50) represents the marginal treatment effect (MTE) for those patients at that particular normalized level of unobserved confounders.

Similarly, for another dyad of NBA, d' and d'+ ε, one can estimate another MTE at another normalized level of unobserved confounder. In this way, a full schedule of MTEs can be estimated that vary over the unobserved confounder levels (i.e. past medical history here) given the level of the observed confounders (i.e. age here). MTEs can be calculated by considering different values of the observed covariates, which will imply different values of the normalized unobserved confounders.[2] Once MTEs are estimated over the range of observed and normalized unobserved confounder levels, they can then be aggregated to form meaningful treatment effect parameters such as the ATE, CATEs, ATT and ATC.

---

[2] In this study, MTEs are estimated at 9,064 unique covariate/IV combinations.

© 2019 Grieve R et al. *JAMA Network Open*.

2.3 Person-centered treatment (PeT) effects

The MTEs can also be aggregated to study heterogeneity in effects using person-centered treatment (PeT) effects. PeT effects are obtained by averaging the MTEs over only the normalized levels of the unobserved confounder (e.g. medical history) that conforms with the observed decision whether or not to transfer the patient to ICU. Intuitively, if based on a patient's observed information (age), it is unlikely that they would be transferred to ICU, but we observe that they are in fact transferred, this conveys useful information about their unobserved confounders (e.g. medical history) (See eFigure 2 for further details). Accordingly, when averaging the MTEs to estimate an effect for this patient conditional on their observed covariates, we would not consider MTEs that imply values of the unobserved confounders that are incompatible with the observed transfer decision. By taking account of the individual's context in this manner, the PeT effect is more personalized than CATEs which average across all of the MTEs conditional on the observed covariates.

We next consider the formal models on which the estimation of PeT effects is based.

eAppendix 3: Formal models behind essential heterogeneity, marginal treatment effects and person-centered treatment (PeT) effects.

3.1 Structural models

We start by formally developing structural models of outcomes and treatment choice [11,12]. There are two treatment states (D = 0 or 1) – transfer to ICU *(treated)* state denoted by *D* = 1 and continued care in a general ward *(untreated)* state denoted by *D* = 0. The corresponding potential individual outcomes (Y_D) in these two states are denoted by Y_1 and Y_0 and can be defined as:

(Eq. 1) $Y_1 = \mu_1(X_o, X_{u_Y}, \varepsilon)$ and $Y_0 = \mu_0(X_o, X_{u_Y}, \varepsilon)$

where X_0 is a vector of observed random variables, X_{u_Y} is a vector of unobserved random variables which are also believed to influence treatment selection (they are the unobserved confounders), and ε is an unobserved random variable that captures all the remaining unobserved random variables which influence outcomes but not treatment selection. We assume that observed covariates are exogenous (assumption 1a and 1b) implying that endogeneity only arises through the decision of whether or not to transfer the patient to ICU (*D*):

© 2019 Grieve R et al. *JAMA Network Open*.

Assumption 1: (a) $(X_o, X_{u_Y}) \perp \varepsilon$ and **(b)** $X_o \perp X_{u_Y}$

We assume the existence of an instrumental variable, Z, that influences whether the patient is transferred to ICU (assumption 2a), but is independent of the unobserved confounders (assumption 2b):

Assumption 2: $(\boldsymbol{a})\ cov(D, Z|X_O) \neq 0$ and **(b)** $X_{u_Y} \perp Z|X_O$

Prior to the realization of the outcome of interest, Y_D, individuals are assigned to be in treatment state 1 or 0 according to whether the influence of the observed covariates and the IV that encourage ICU transfer, dominate the influence of the unobserved confounders (X_{u_D}) that discourage ICU transfer as represented in equation 2:

(Eq. 2) $D = 1$ if $\mu_D(X_o, Z) - X_{u_D} > 0$, and D = 0 otherwise

where μ_D is an unknown function of X_0 and Z, and X_{u_D} is a random variable that captures X_{u_Y} and all remaining unobserved random variables influencing the transfer decision (but not outcomes).

Equations (**Eq. 1**) and (**Eq. 2**) represent the nonparametric models that conform to the Imbens and Angrist's independence and monotonicity assumptions [19] needed to interpret instrumental variable estimates in a model of heterogeneous returns [13]. While Equation 2 is written in levels, we can re-express it in terms of probabilities. As in Heckman and Vytlacil (1999, 2001, 2005)[11-13], we can rewrite **(Eq. 2)** as

(Eq. 3) $D = 1$ if $P(X_o = x_o, Z = z) > V$, and D=0 otherwise

where $V = F_{X_{u_D}}[X_{u_D}|X_o = x_o, Z = z]$, $P(x_o, z) = F_{X_{u_D}|x_o,z}[\mu_D(x_o, z)|X_o, Z]$ and F represents a cumulative distribution function. Therefore, for any arbitrary distribution of X_{u_D} conditional on X_O and Z, by definition, $V \sim$ Uniform[0, 1] conditional on X_O and Z.

To estimate the probability of being in treatment state 1 consistently requires that the instrument is conditionally independent of the unobserved covariates influencing the transfer decision (Assumption 3):

Assumption 3: $X_{u_D} \perp Z|X_O$

© 2019 Grieve R et al. *JAMA Network Open*.

3.2 Marginal Treatment Effects

A MTE is perhaps the most nuanced estimable effect. It identifies an effect for an individual whose observed (x_O and z) and unobserved covariates (captured by v which includes x_{u_Y}) make his physician indifferent between transferring the patient to ICU or keeping them in the general ward i.e. $P(x_o, z) = v$. Under regular IV assumptions, Heckman and Vytlacil (2001) [12] show that Marginal Treatment Effects can be identified by

$$\textbf{(Eq. 4)}\quad \frac{\partial E_\varepsilon(Y|x_o,Z)}{\partial p} = E_\varepsilon\big((Y_1 - Y_0)\big|X_o, V = v\big) = MTE(x_o, v),$$

where Y_1 and Y_0 are the outcomes in State 1 and 0, Y = $D*Y_1$ + (1 - D)*Y_0 is the observed outcome and p is the propensity score.

3.3 Person Centered Treatment Effects

For a particular individual we will not observe v, meaning we cannot estimate their treatment effect $E(Y_1 - Y_0|x_o, v)$ and they may not be marginal (i.e. $P(x_o, z) \neq v$) making the MTE inappropriate. However, their actual treatment assignment allows us to infer that $v < P(x_o,z)$ if we observe they were transferred to ICU and $v \geq P(x_o,z)$ if they remained in a general ward. This insight allowed Basu (2014) [16] to define the Person centered treatment (PeT) effect as $E(Y_1 - Y_0|x_o, V < P(x_o, z))$ for individuals in the treated group (ICU) and $E(Y_1 - Y_0|x_o, V \geq P(x_o, z))$ for individuals in the control group (general wards). The PeT is more nuanced than the CATE as it takes account of the plausible range of values that v may take.

Conceptually, a PeT effect is a weighted average of MTEs. For an individual in the treated group, the PeT effect would be[3]:

$$PeT\ \ effect = E\big(Y_1 - Y_0\big|x_o, V < P(x_o, z)\big) = P(x_o, z)^{-1}\int_0^{P(x_o,Z)} MTE(x_o, v)\, dv$$

For any given individual, the PeT effect identifies the specific margins where that individual may belong given their individual values of X_O, $P(x_o, z)$ and D. It then averages the MTEs over those margins. This distinguishes the PeT from the CATE which averages MTEs across all margins.

[3] Similarly, a PeT effect for a person in the control group can be obtained by integrating MTEs over values of V greater than $P(x_o, z)$.

© 2019 Grieve R et al. *JAMA Network Open*.

eAppendix 4: Estimating marginal treatment effects (MTEs) and PeT effects.

Before estimating PeT effects, we must first estimate the MTEs using an LIV approach. A control function is estimated, which models how the outcome (Y) varies over:

I. the observed patient and ICU-level characteristics (risk factors)
II. the estimated IV-dependent propensity to be transferred to ICU ($\hat{p}(x_o, z)$) and interactions between the propensity score and the risk factors and
III. polynomials of the propensity score ($K(\alpha; \hat{p}(x_o, z))$).

This control function can be written in general form as:

(Eq. 5) $$\mathrm{E}(Y|X_O, \hat{p}(x,z)) = g\left(\alpha_0 + \alpha_1 X_O + \alpha_3 \times \hat{p}(x_o, z) \times X_O + K\big(\alpha; \hat{p}(x_o, z)\big)\right)$$

where g is a function that depends on the properties of the outcome variable (e.g. the normal or logistic CDF for a binary variable). The partial derivate of the outcome, as characterized by the control function, with respect to the IV-dependent propensity score, $\hat{p}(x_o, z)$ (reflecting marginal changes in the IV), estimates the marginal treatment effect according to specific values of the scalar unobserved risk factor levels ($V = \hat{p}(x_o, z)$).

$$MTE = \frac{\partial g\left(\alpha_0 + \alpha_1 X_O + \alpha_3 \times \hat{p}(x_o, z) \times X_O + K\big(\alpha; \hat{p}(x_o, z)\big)\right)}{\partial p}$$

To calculate the PeT estimates, the MTE is calculated at different possible values of $\hat{p}(x_o, z)$ corresponding to different possible values at which the patient would be a marginal patient (i.e. $\hat{p}(x_o, z) = V$). Next the MTEs that correspond to values of V that would be inconsistent with whether or not the patient was actually transferred to ICU (based on **Eq. 3**) are eliminated. The remaining MTEs are then averaged to give the patient's PeT estimate.

eAppendix 5: Implementation of PeT for evaluating the effectiveness of ICU transfer for deteriorating ward patients.

To implement the PeT method on the matched sample, we first estimate the probability of being in the treated group (D=1) conditional on the covariates and the IV (number of ICU bed available) and predict the propensity score for each individual in the matched sample $\hat{P}(x_o, z)$. We require that this propensity score has mass at any value (rounded to 0.01) for both levels of the exposure so observations at values of the rounded propensity score that do not meet this criterion are dropped.

© 2019 Grieve R et al. *JAMA Network Open*.

Next we determine an appropriate model for the outcome equation, g(.). Since our outcome of interest, mortality, is binary we use a probit model for this equation also. We include the baseline covariates, hospital fixed effects, the propensity score and also include interactions between the covariates and the propensity score as suggested in Basu (2015) [17]. The hospital fixed effects are not interacted with the propensity score to preserve degrees of freedom.

After specifying this equation, which is the second stage of an LIV estimand (Eq. 5), we obtain marginal treatment effects using numerical integration. To do so, we compute the marginal treatment effect $\left(\frac{d\hat{g}}{d\hat{p}}\right)$, and then evaluate it 1,000 times replacing $\hat{p}$ by a random draw, u, from a uniform distribution with the minimum and maximum values determined by the range of the estimated propensity scores. These values represent the distribution of MTEs for the individual at different possible levels of unobserved covariates, V. Next, we determine for each of the 1,000 draws of u, whether the value would be consistent with the individual being in the treated or comparison group as in Equation 2 above. To do so we define the latent variable $D^* = \left\{\Phi^{-1}\left(\hat{P}(x_o, z)\right) + \Phi^{-1}(1-u)\right\}$, with the individual assigned to treatment if D* > 0 and assigned to the comparison group otherwise. The PeT for the individual is then calculated by averaging the MTEs over the subset of MTEs consistent with their actual treatment assignment.

These individual level PeTs are then aggregated to obtain a CATE for a number of subgroups: for the treated group giving the average treatment effect on the treated (ATT), for the comparison group giving the average treatment effect on the untreated (ATUT) and also by age category, NEWS score, NEWS risk category, ICNARC score, SOFA score, and age category combined with each of the physiology measures.

To obtain standard errors for the PeT effect estimates, we use a bootstrap approach, where we resample with replacement 1,000 times and for each bootstrap sample we repeat the entire process outlined above including estimation of the propensity score and the outcome models. The standard deviation of the individuals' (or sub groups') bootstrapped estimates represents the standard error of the PeT estimate.

eAppendix 6: Details of predictive models.

We applied a logistic regression analysis to explore which subgroups of patients were predicted to have an estimated reduction in the absolute risk of 28-day mortality following ICU transfer of greater

© 2019 Grieve R et al. *JAMA Network Open*.

(or less) than 10%, the magnitude of clinical benefit upon which the initial power calculation for the (SPOT)light study was based. Of the 9,068 patients, 3,472 (38.5%) had PeT estimates exceeding this threshold (PeT < -10%). The model included the following predictors: age, age squared, gender, diagnosis of sepsis, peri-arrest, NEWS, SOFA and ICNARC physiology scores, CCMDS level at assessment and recommended level, and timing (out of hours, winter, and weekend or not). Since PeT estimates for some patients are more precisely estimated than for others, the logistic regression was also estimated after weighting each individual's data by the inverse of the standard error of their PeT estimate. The results are presented in eTable 6. The patients predicted to benefit more from ICU transfer were those who were older, admitted during winter, during the week and within usual office hours, and with higher physiology scores indicating greater severity.

eAppendix 7: Sensitivity Analyses.

As an initial sensitivity check, the ATE calculated by averaging the individual PeT estimates was compared to estimates obtained from two stage least squares (2SLS). 2SLS does not estimate the ATE, rather it estimates a weighted average of local average treatment effect (LATE). We note that the LATE estimate is larger than the PeT effects (eTable 3), although it is not immediately clear to which patients this estimate relates.eTables 10 and 11 compare average treatment effect estimates based on alternative model specifications to the base case. In the baseline model (Model 1), the treatment and outcome equations were modelled using probit functional form. To assess whether results are sensitive to the functional form assumption, Model 2 used a logistic regression for the treatment equation and Model 3 used logistic regression for both the treatment and outcome equations. To assess the sensitivity of results to the inclusion/exclusion of explanatory variables Model 4 removed the physiology scores completely, Model 5 included the physiology scores but did not include squared terms. Model 6 included interactions between the physiology scores but did not include squared terms, while Model 7 included physiology scores, squared terms and interactions. As shown in eTable 10, the overall ATE is quite robust to these changes. eTable 11 reports similar sensitivity analyses for the conditional average treatment effects by subgroups for 90 day mortality. The subgroups are, as described above, based on the type of care (general ward vs ICU), age category, NEWS risk category and NEWS score. The subgroups effects are robust to changes to the model specification, although leaving out the physiology score alters results somewhat as might be expected (Model 4).

© 2019 Grieve R et al. *JAMA Network Open*.

eReferences

1. Baiocchi M, Small DS, Lorch S, Rosenbaum PR. Building a stronger instrument in an observational study of perinatal care for premature infants. *J Am Stat Assoc.* 2010;105(492):1285-1296.
2. Baiocchi M, Small DS, Yang L, Polsky D, Groeneveld PW. Near/far matching: a study design approach to instrumental variables. *Health Serv Outcomes Res Methodol.* 2012;12(4):237-253.
3. Lorch SA, Baiocchi M, Ahlberg CE, Small DS. The differential impact of delivery hospital on the outcomes of premature infants. *Pediatrics.* 2012:peds. 2011-2820.
4. Keele LJ, Harris S, Grieve RD. Stronger instruments and refined covariate balance in an observational study of the effectiveness of prompt admission to the ICU in the UK. Journal of The Royal Statistical Society, Series A. 2019; In Press.
5. Rosenbaum PR. *Design of Observational Studies.* New York: Springer-Verlag; 2010.
6. Rosenbaum PR. Optimal matching of an optimally chosen subset in observational studies. *J Comput Graph Stat.* 2012;21(1):57-71.
7. Valley TS, Sjoding MW, Ryan AM, Iwashyna TJ, Cooke CR. Association of intensive care unit admission with mortality among older patients with pneumonia. *JAMA.* 2015;314(12):1272-1279.
8. Harris KM, Remler DK. Who is the marginal patient? Understanding instrumental variables estimates of treatment effects. *Health Serv Res.* 1998;33(5 Pt 1):1337.
9. McClellan M, McNeil BJ, Newhouse JP. Does more intensive treatment of acute myocardial infarction in the elderly reduce mortality?: analysis using instrumental variables. *JAMA.* 1994;272(11):859-866.
10. Newhouse JP, McClellan M. Econometrics in outcomes research: the use of instrumental variables. *Annu Rev Public Health.* 1998;19(1):17-34.
11. Heckman JJ, Vytlacil EJ. Local instrumental variables and latent variable models for identifying and bounding treatment effects. *Proceedings of the national Academy of Sciences.* 1999;96(8):4730-4734.
12. Heckman JJ, Vytlacil E. Local instrumental variables. In: Hsiao C, Morimue K, Powell J, eds. *Nonlinear Statistical Modeling: Proceedings of the Thirteenth International Symposium in Economic Theory and Econometrics: Essays in Honor of Takeshi Amemiya.* Vol 13: Cambridge University Press; 2001:1-46.
13. Heckman JJ, Vytlacil E. Structural equations, treatment effects, and econometric policy evaluation 1. *Econometrica.* 2005;73(3):669-738.
14. Klotz L. Prostate cancer overdiagnosis and overtreatment. *Curr Opin Endocrinol Diabetes Obes.* 2013;20(3):204-209.
15. Swanson SA, Hernán MA. Think globally, act globally: an epidemiologist's perspective on instrumental variable estimation. *Stat Sci.* 2014;29(3):371.
16. Basu A. Estimating Person-Centered Treatment (Pet) Effects Using Instrumental Variables: An Application to Evaluating Prostate Cancer Treatments. *J Appl Econ (Chichester Engl).* 2014;29(4):671-691.
17. Basu A. Person-centered treatment (PeT) effects: Individualized treatment effects using instrumental variables. *Stata J.* 2015:397.
18. Basu A, Gore JL. Are elderly patients with clinically localized prostate cancer overtreated? Exploring heterogeneity in survival effects. *Med Care.* 2015;53(1):79.
19. Angrist J, Imbens G. Identification and estimation of local average treatment effects. *Econometrica.* 1994;62:467-476.

© 2019 Grieve R et al. *JAMA Network Open*.

Supplementary Figures and Tables

eTable 1: Baseline characteristics of all deteriorating ward patients receiving ICU versus General Ward care, before matching.

Characteristics	ICU		General ward		Standardized difference
No. of admissions	4,994		8,017		
No. of ICU beds available					
Mean (SD)	4.63	(3.22)	4.05	(3.13)	0.181
Median (Min, Max)	4	(0, 18)	3	(0, 19)	
Age, mean (SD)	63.77	(16.74)	66.08	(18.30)	-0.132
Male sex, n (%)	2,728	(54.6%)	4,105	(51.2%)	0.069
Reported sepsis diagnosis, n (%)	3,380	(67.7%)	4,553	(56.8%)	0.226
CCMDS level of care at visit, n (%)					
Level 0	490	(9.8%)	1,231	(15.4%)	-0.168
Level 1	3,064	(61.4%)	5,774	(72.0%)	-0.228
Level 2	1,301	(26.1%)	944	(11.8%)	0.371
Level 3	104	(2.1%)	22	(0.3%)	0.168
Missing	35	(0.7%)	46	(0.6%)	0.016
Recommended CCMDS level of care at visit, n (%)					
Level 0	86	(1.7%)	838	(10.5%)	-0.371
Level 1	1,183	(23.7%)	5,830	(72.7%)	-1.126
Level 2	2,539	(50.8%)	1,229	(15.3%)	0.815
Level 3	1,152	(23.1%)	52	(0.6%)	0.739
Missing	34	(0.7%)	68	(0.8%)	-0.019
Peri-arrest, n (%)	456	(9.1%)	191	(2.4%)	0.293
Acute Physiology scores, mean (SD)					
ICNARC	17.40	(7.76)	13.63	(6.55)	0.524
SOFA	3.90	(2.33)	2.68	(1.95)	0.565
NEWS	7.07	(3.17)	5.68	(2.93)	0.456
NEWS Risk class, n (%)					
None	93	(1.9%)	258	(3.2%)	-0.086
Low	956	(19.1%)	2,451	(30.6%)	-0.267
Medium	1,240	(24.8%)	2,487	(31.0%)	-0.138
High	2,705	(54.2%)	2,821	(35.2%)	0.389
Time of admission, n (%)					
Weekend	1,261	(25.3%)	1,969	(24.6%)	0.016
Out of hours	1,983	(39.7%)	2,608	(32.5%)	0.150
Winter	1,299	(26.0%)	2,064	(25.7%)	0.006

ICU, intensive care unit; SD, Standard Deviation, CCMDS, Critical Care Minimum Dataset; ICNARC ICNARC, Intensive Care National Research and Audit Centre; SOFA, Sequential Organ Failure Assessment; NEWS, National Early Warning Score. The NEWS score ranges from 0 (least severe) to 20 (most severe). The SOFA score from 0 (least severe) to 14 (most severe), and the ICNARC physiology score from 0 (least severe) to 100 (most severe).

© 2019 Grieve R et al. *JAMA Network Open*.

eTable 2: Baseline characteristics for the patients in the unmatched versus matched samples.

Characteristics	Unmatched		Matched	
Number of admissions	13,011		9,192	
Age	65.19	(17.8)	65.11	(17.5)
Male, n (%)	6,833	(52.5%)	4,874	(53.0%)
Reported sepsis diagnosis (any), n (%)	7,933	(61.0%)	5,741	(62.5%)
CCMDS level of care at visit, n (%)				
Level 0	1,721	(13.2%)	1,100	(12.0%)
Level 1	8,838	(67.9%)	6,409	(69.7%)
Level 2	2,245	(17.3%)	1,547	(16.8%)
Level 3	126	(1.0%)	84	(0.9%)
Missing	81	(0.6%)	52	(0.6%)
Peri-arrest, n (%)	647	(5.0%)	397	(4.3%)
Acute Physiology scores				
ICNARC	15.08	(7.28)	15.15	(7.26)
SOFA	3.15	(2.18)	3.15	(2.17)
NEWS	6.21	(3.10)	6.23	(3.08)
NEWS Risk class, n (%)				
None	351	(2.7%)	245	(2.7%)
Low	3,407	(26.2%)	2,394	(26.0%)
Medium	3,727	(28.6%)	2,591	(28.2%)
High	5,526	(42.5%)	3,962	(43.1%)
Time of admission				
Weekend	3,230	(24.8%)	2,231	(24.3%)
Out of hours	4,591	(35.3%)	3,198	(34.8%)
Winter	3,363	(25.8%)	1,920	(20.9%)

ICU, intensive care unit; SD, Standard Deviation, CCMDS, Critical Care Minimum Dataset; ICNARC ICNARC, Intensive Care National Research and Audit Centre; SOFA, Sequential Organ Failure Assessment; NEWS, National Early Warning Score. The NEWS score ranges from 0 (least severe) to 20 (most severe). The SOFA score from 0 (least severe) to 14 (most severe), and the ICNARC physiology score from 0 (least severe) to 100 (most severe).

© 2019 Grieve R et al. *JAMA Network Open*.

eTable 3: Comparison of overall effects, estimated by PeT versus 2-stage least squares (2LS).

	Baseline PeT Model	2 SLS
**28-day mortality**	-4.9%	-34.00%
	(-26.4%, 16.6%)	(-89.9%, 21.9%)
**90-day mortality**	-4.7%	-25.60%
	(-28.5%, 19.2%)	(-83.8%, 32.5%)

© 2019 Grieve R et al. *JAMA Network Open*.

eTable 4: Summary of effects on 28-day mortality, by subgroup.

	Incremental effect***	95% Confidence Interval	
**Type of care:**			
General ward	3.3%	-15.2%	21.8%
ICU	-10.1%	-33.2%	13.0%
**Age category**			
18-23	7.7%	-5.5%	21.0%
24-29	7.1%	-6.7%	21.0%
30-35	5.9%	-7.7%	19.4%
36-41	3.0%	-12.3%	18.2%
42-47	1.9%	-13.4%	17.2%
48-53	0.1%	-16.0%	16.2%
54-59	-2.4%	-21.0%	16.2%
60-65	-3.7%	-22.9%	15.5%
66-71	-4.1%	-24.4%	16.1%
72-77	-5.0%	-26.5%	16.6%
78-83	-4.2%	-26.9%	18.5%
84-89	-1.9%	-25.9%	22.1%
90-95	2.6%	-24.2%	29.3%
96-101	8.5%	-22.1%	39.0%
**NEWS risk category**			
0	3.2%	-12.4%	18.9%
1	2.4%	-14.1%	18.9%
2	-0.2%	-19.2%	18.9%
3	-5.8%	-28.0%	16.3%

	Incremental effect***	95% Confidence Interval	
**NEWS score**			
0	3.7%	-12.1%	19.5%
1	2.8%	-12.9%	18.4%
2	4.0%	-11.8%	19.7%
3	2.9%	-14.1%	20.0%
4	2.7%	-14.1%	19.5%
5	1.1%	-17.5%	19.7%
6	-0.9%	-20.5%	18.7%
7	-1.1%	-21.5%	19.3%
8	-3.9%	-25.4%	17.6%
9	-5.6%	-28.2%	17.0%
10	-8.4%	-31.6%	14.7%
11	-11.2%	-35.7%	13.2%
12	-16.6%	-43.7%	10.5%
13	-13.6%	-38.6%	11.4%
14	-20.6%	-51.4%	10.2%
15	-22.7%	-55.2%	9.7%
16	-33.7%	-80.0%	12.5%
17	-25.0%	-59.7%	9.6%
18	-19.6%	-74.0%	34.8%
19	-25.4%	-50.6%	-0.2%

© 2019 Grieve R et al. *JAMA Network Open*.

eTable 5: Summary of effects on 28- and 90-day mortality, by age group and level of NEWS score.

	28-day mortality			90-day mortality		
	**Incremental effect*** **	**95% Confidence Interval**		**Incremental effect*** **	**95% Confidence Interval**	
**Aged < 75 & NEWS score:**						
Low (<5)	1.4%	-14.5%	17.4%	2.5%	-18.0%	23.0%
Moderate (5-6)	-2.1%	-21.1%	16.9%	-1.1%	-23.0%	20.8%
High (>6)	-8.4%	-31.0%	14.1%	-6.8%	30.8%	17.3%
**Aged ≥ 75 & NEWS score:**						
Low (<5)	-1.0%	-24.8%	22.8%	-3.3%	-31.6%	25.0%
Moderate (5-6)	-4.8%	-30.5%	20.9%	-7.0%	-34.0%	20.1%
High (>6)	-11.6%	-39.0%	15.8%	-12.5%	-40.0%	15.1%

© 2019 Grieve R et al. *JAMA Network Open*.

eTable 6: Baseline characteristics explaining variations in PeT effects following ICU transfer versus general ward care on 28-day mortality.

	Unweighted		Weighted by inverse of standard deviation of PeT	
	**Odds Ratio**	**95% CI**	**Odds Ratio**	**95% CI**
Age	1.144***	(1.091, 1.2)	1.243**	(1.153, 1.34)
Age squared	0.999***	(0.999, 1)	0.999***	(0.998, 0.999)
Male	0.904	(0.795, 1.029)	0.848**	(0.746, 0.965)
Reported sepsis diagnosis (any)	0.641***	(0.536, 0.766)	0.618***	(0.506, 0.756)
CCMDS level of care at visit				
Level 1	0.061***	(0.031, 0.123)	0.036***	(0.013, 0.095)
Level 2	0.430**	(0.224, 0.827)	0.28**	(0.101, 0.778)
Level 3	0.789	(0.238, 2.609)	0.429	(0.072, 2.546)
Missing	0.359**	(0.151, 0.851)	0.551	(0.14, 2.165)
Peri-arrest	3.631***	(2.46, 5.359)	2.631***	(1.792, 3.863)
Acute Physiology scores				
ICNARC	1.193***	(1.143, 1.245)	1.254***	(1.191, 1.32)
SOFA	1.083***	(1.064, 1.102)	1.088***	(1.059, 1.118)
NEWS	2.113***	(1.868, 2.389)	2.324***	(1.958, 2.758)
Time of admission				
Weekend	0.752***	(0.611, 0.926)	0.591***	(0.464, 0.753)
Out of hours	0.404***	(0.31, 0.528)	0.395***	(0.249, 0.627)
Winter	1.133	(0.913, 1.406)	1.353*	(0.964, 1.899)

Reference group: Reduction in absolute risk of 28-day mortality following ICU transfer of less than 10%, Statistically significantly different from 1: *** = at 1% level; **=at 5% level; * = at 10% level.

© 2019 Grieve R et al. *JAMA Network Open*.

eTable 7: Local instrumental variable (LIV) model for 28-day mortality.

	Main effect		Interaction with propensity score	
	**Odds Ratio**	**95% Confidence Interval**	**Odds Ratio**	**95% Confidence Interval**
Age	-0.033	(-0.08, 0.014)	0.034***	(0.011, 0.058)
Age squared	0.0002	(-0.0002, 0.001)	-0.0001	(-0.0003, 0.0001)
Male	0.003	(-0.233, 0.239)	0.022	(-0.088, 0.133)
Reported sepsis diagnosis (any)	0.09	(-0.135, 0.315)	-0.03	(-0.147, 0.087)
**CCMDS level of care at visit**				
Level 2	-0.191	(-0.534, 0.151)	0.113	(-0.123, 0.348)
Level 3	2.557	(-18.1, 23.2)	-2.144	(-22.8, 18.5)
Missing	0.854	(-58.1, 59.8)	-0.108	(-27.2, 27.0)
**Recommended CCMDS level of care at visit**				
Level 2	0.034	(-0.607, 0.675)	0.122	(-0.336, 0.58)
Level 3	-0.655	(-4.177, 2.867)	0.962	(-2.784, 4.708)
Missing	-8.295	(-352.9, 336.3)	0.492	(-16.7, 17.7)
Peri-arrest	-0.052	(-0.541, 0.436)	-0.072	(-0.45, 0.305)
**Acute Physiology scores**				
ICNARC	-0.0005	(-0.061, 0.06)	0.015	(-0.025, 0.056)
ICNARC-squared	-0.0002	(-0.002, 0.001)	0.0002	(-0.001, 0.001)
NEWS	0.001	(-0.114, 0.115)	0.058*	(-0.008, 0.123)
NEWS-squared	-0.001	(-0.009, 0.006)	0.00002	(-0.004, 0.005)
SOFA	-0.129	(-0.321, 0.063)	-0.001	(-0.097, 0.095)
SOFA-squared	0.003	(-0.016, 0.022)	0.014**	(0.002, 0.025)
**Time of admission**				
Out of hours	0.147	(-0.065, 0.358)	-0.013	(-0.127, 0.101)
Weekend	0.178	(-0.069, 0.425)	-0.039	(-0.161, 0.082)
Winter	-0.01	(-0.278, 0.259)	-0.002	(-0.146, 0.141)
Constant	-3.49***	(-4.181, -2.8)	1.614*	(-0.185, 3.413)

Statistically significantly different from 1: *** = at 1% level; **=at 5% level; * = at 10% level.
Note: Control for hospital were also included but are not shown.
Test of joint statistical significance of interactions: chi-square = 45.67; p-value = 0.0014

© 2019 Grieve R et al. *JAMA Network Open*.

eTable 8: 90-day Mortality following ICU versus General Ward care for the matched sample.

	Sample size*	ICU, Deaths N** (%)	General Ward, Deaths N** (%)	Risk Difference***	[95% CI]****
**Estimator**					
IV (PeT, Logit)	9,015	2660 (29.5%)	3079 (34.2%)	-4.7%	(-28.5% to 19.2%)
IV (PeT, Probit)	9,015	2598 (28.3%)	2864 (31.1%)	-2.5%	(-23.7% to 18.7%)
Regression	9,192	3094 (33.7%)	2556 (27.8%)	5.9%	(3.3% to 8.4%)
Unadjusted	9,192	3357 (36.5%)	2394 (26.0%)	10.5%	(8.5% to 12.4%)

*For each method, the maximum sample size was 9,192. Observations were excluded if there is not mass at any value (rounded to 0.01) of the propensity score for both levels of exposure as recommended by Basu (2015). **The number of predicted deaths is rounded to the nearest whole number. *** Difference in percentage of deaths from the PeT Instrumental variable estimate. *** Normal based CI with SE calculated with the non-parametric bootstrap allowing for clustering by hospital. *** difference in percentage of deaths from the PeT Instrumental variable estimate ** Normal based CI with SE calculated with the non-parametric bootstrap allowing for clustering by hospital

ICU, intensive care unit; CI, confidence interval; IV, Instrumental Variable; PeT, Person-centred treatment effect;

© 2019 Grieve R et al. *JAMA Network Open*.

eTable 9: Summary of effects of ICU transfer on 90-day mortality, by subgroup.

	Incremental effect***	95% Confidence Interval	
**Type of care:**			
General ward	2.6%	-18.4%	23.6%
ICU	-10.7%	-32.8%	11.4%
**Age category**			
18-23	11.1%	-4.2%	26.5%
24-29	10.4%	-5.6%	26.3%
30-35	8.2%	-7.2%	23.7%
36-41	4.5%	-12.5%	21.5%
42-47	3.2%	-13.8%	20.3%
48-53	0.6%	-17.3%	18.5%
54-59	-2.0%	-22.1%	18.1%
60-65	-4.1%	-24.7%	16.5%
66-71	-5.2%	-26.8%	16.5%
72-77	-6.4%	-28.8%	16.1%
78-83	-6.4%	-29.7%	16.9%
84-89	-4.4%	-28.7%	19.8%
90-95	-1.0%	-27.9%	25.8%
96-101	4.3%	-24.5%	33.0%
**NEWS risk category**			
0	4.4%	-17.1%	25.9%
1	2.1%	-17.6%	21.8%
2	-0.9%	-21.3%	19.4%
3	-6.7%	-28.5%	15.2%

	Incremental effect***	95% Confidence Interval	
**NEWS score**			
0	4.9%	-17.0%	26.9%
1	3.0%	-17.2%	23.2%
2	3.3%	-16.4%	23.0%
3	2.7%	-17.6%	23.1%
4	1.9%	-17.3%	21.1%
5	0.4%	-19.9%	20.7%
6	-2.1%	-22.5%	18.4%
7	-2.3%	-23.4%	18.8%
8	-5.2%	-26.5%	16.1%
9	-6.4%	-28.4%	15.5%
10	-9.3%	-31.6%	13.0%
11	-11.6%	-35.2%	12.1%
12	-15.3%	-41.3%	10.7%
13	-12.0%	-36.6%	12.5%
14	-17.2%	-46.7%	12.4%
15	-18.7%	-48.8%	11.5%
16	-27.6%	-71.3%	16.1%
17	-17.0%	-51.7%	17.7%
18	-17.9%	-63.5%	27.7%
19	-19.2%	-40.4%	1.9%

© 2019 Grieve R et al. *JAMA Network Open*.

eTable 10: Sensitivity analysis of conditional average treatment effects on 28-day mortality, overall, and by subgroup.

	Baseline PeT Model 1		Model 2		Model 3		Model 4		Model 5		Model 6		Model 7	
	Incremental effect***	95% CI	Incremental effect***	95% CI	Incremental effect***	95% CI	Incremental effect***	95% CI	Incremental effect***	95% CI	Incremental effect***	95% CI	Incremental effect***	95% CI
**Overall average**	-4.9%	(-26.4%, 16.6%)	-1.8%	(-21%, 17.3%)	-2.3%	(-21.4%, 16.7%)	-1.4%	(-23.6%, 20.8%)	-3.1%	(-20.2%, 14.0%)	-5.1%	(-24.5%, 14.2%)	-2.0%	(-21.5%, 17.5%)
**Type of care:**														
General ward	-0.4%	(-21%, 20.3%)	3.3%	(-15.1%, 21.7%)	3.1%	(-15.3%, 21.6%)	1.9%	(-20.1%, 23.9%)	1.8%	(-14.2%, 17.9%)	0.3%	(-18.3%, 19.0%)	3.0%	(-15.7%, 21.7%)
ICU	-12.6%	(-37.2%, 12%)	-10.1%	(-32.4%, 12.3%)	-11.0%	(-33.0%, 10.9%)	-6.7%	(-31.7%, 18.3%)	-11.0%	(-32.1%, 10.2%)	-14.1%	(-36.3%, 8.2%)	-10.1%	(-33.0%, 12.8%)
**Age category**														
18-23	5.5%	(-8.3%, 19.2%)	7.7%	(-5.9%, 21.3%)	9.0%	(-4.5%, 22.5%)	7.1%	(-7.8%, 22.0%)	6.6%	(-5.6%, 18.9%)	5.0%	(-8.0%, 18.0%)	7.4%	(-6.6%, 21.4%)
24-29	5.0%	(-9.9%, 19.9%)	7.1%	(-6.9%, 21.2%)	8.7%	(-5.2%, 22.5%)	6.8%	(-8.6%, 22.1%)	6.0%	(-6.7%, 18.7%)	4.1%	(-9.6%, 17.9%)	6.8%	(-7.6%, 21.3%)
30-35	3.6%	(-11%, 18.2%)	5.8%	(-7.7%, 19.4%)	7.1%	(-6.1%, 20.4%)	5.5%	(-9.9%, 20.8%)	4.5%	(-7.5%, 16.6%)	2.9%	(-10.2%, 16.1%)	5.5%	(-8.5%, 19.4%)
36-41	0.9%	(-15.4%, 17.1%)	3%	(-12.0%, 18.1%)	4.1%	(-10.5%, 18.8%)	3.5%	(-13.6%, 20.6%)	2.4%	(-11.3%, 16.0%)	0.4%	(-14.1%, 15.0%)	2.8%	(-12.7%, 18.3%)
42-47	-0.5%	(-17.3%, 16.2%)	1.8%	(-13.4%, 17.0%)	2.8%	(-12.1%, 17.7%)	2.1%	(-15.3%, 19.5%)	0.9%	(-12.7%, 14.5%)	-1.0%	(-16.2%, 14.2%)	1.9%	(-13.6%, 17.4%)
48-53	-1.8%	(-19.2%, 15.7%)	0.3%	(-15.6%, 16.2%)	0.7%	(-14.9%, 16.2%)	0.5%	(-18.3%, 19.2%)	-0.7%	(-15.1%, 13.7%)	-2.3%	(-18.1%, 13.6%)	0.0%	(-16.2%, 16.1%)
54-59	-5.0%	(-25.4%, 15.5%)	-2.5%	(-20.9%, 15.9%)	-2.6%	(-20.7%, 15.6%)	-1.9%	(-23.2%, 19.4%)	-3.4%	(-20.0%, 13.3%)	-5.8%	(-23.9%, 12.4%)	-2.5%	(-21.2%, 16.2%)
60-65	-6.3%	(-27.4%, 14.8%)	-3.6%	(-22.4%, 15.2%)	-4.0%	(-22.7%, 14.7%)	-2.6%	(-24.7%, 19.5%)	-4.5%	(-21.5%, 12.6%)	-6.5%	(-25.3%, 12.2%)	-3.7%	(-22.8%, 15.4%)
66-71	-7.2%	(-29.8%, 15.4%)	-4.1%	(-24.2%, 15.9%)	-5.0%	(-25.1%, 15.0%)	-3.6%	(-26.9%, 19.7%)	-5.4%	(-23.4%, 12.7%)	-7.5%	(-27.9%, 12.8%)	-4.4%	(-24.8%, 15.9%)
72-77	-8.2%	(-32.1%, 15.7%)	-4.9%	(-26.2%, 16.3%)	-6.1%	(-27.4%, 15.1%)	-4.0%	(-28.7%, 20.8%)	-6.1%	(-25.2%, 13.0%)	-8.2%	(-29.6%, 13.3%)	-5.2%	(-26.8%, 16.3%)
78-83	-8.0%	(-33.4%, 17.5%)	-4.2%	(-26.7%, 18.4%)	-5.5%	(-28.2%, 17.1%)	-3.3%	(-28.8%, 22.3%)	-5.7%	(-25.7%, 14.3%)	-7.8%	(-30.8%, 15.3%)	-4.4%	(-27.1%, 18.4%)
84-89	-6.3%	(-33%, 20.5%)	-2%	(-25.7%, 21.8%)	-3.3%	(-27.1%, 20.5%)	-1.9%	(-28.8%, 24.9%)	-3.9%	(-25.1%, 17.2%)	-5.9%	(-30.2%, 18.4%)	-2.0%	(-26.1%, 22.2%)
90-95	-2.5%	(-32.2%, 27.2%)	2.6%	(-23.8%, 28.9%)	1.4%	(-25.1%, 27.9%)	0.8%	(-28.5%, 30.1%)	0.2%	(-23.1%, 23.5%)	-1.9%	(-28.9%, 25.2%)	2.7%	(-24.2%, 29.6%)
96-101	3.2%	(-31.5%, 37.9%)	8.5%	(-22.1%, 39.0%)	7.8%	(-23.0%, 38.5%)	5.3%	(-26.3%, 36.9%)	4.4%	(-23.0%, 31.7%)	1.9%	(-29.9%, 33.6%)	8.4%	(-23.0%, 39.8%)
**NEWS risk category**														
**0**	1.5%	(-15.9%, 18.9%)	3.3%	(-11.9%, 18.4%)	3.7%	(-11.1%, 18.6%)	0.3%	(-19.8%, 20.4%)	1.7%	(-11.0%, 14.3%)	2.1%	(-14.8%, 18.9%)	2.7%	(-13.4%, 18.9%)

© 2019 Grieve R et al. *JAMA Network Open*.

**1**	-0.2%	(-18.7%, 18.4%)	2.4%	(-13.9%, 18.7%)	2.4%	(-13.6%, 18.5%)	0.4%	(-20.3%, 21.0%)	0.8%	(-13.2%, 14.8%)	0.1%	(-17.1%, 17.3%)	2.4%	(-14.5%, 19.3%)
**2**	-3.2%	(-24.4%, 18%)	-0.2%	(-19.1%, 18.8%)	-0.6%	(-19.4%, 18.2%)	-0.5%	(-22.3%, 21.3%)	-1.5%	(-18.0%, 15.0%)	-3.7%	(-22.6%, 15.2%)	-0.3%	(-19.4%, 18.8%)
**3**	-9.4%	(-33.6%, 14.8%)	-5.8%	(-27.6%, 16.1%)	-6.7%	(-28.5%, 15.2%)	-3.1%	(-27%, 20.8%)	-6.7%	(-26.9%, 13.4%)	-9.6%	(-31.1%, 11.9%)	-6.0%	(-28.1%, 16.1%)
**NEWS score**														
0	1.7%	(-16%, 19.4%)	3.7%	(-11.6%, 19.0%)	4.2%	(-10.8%, 19.2%)	0.3%	(-19.8%, 20.4%)	1.9%	(-10.6%, 14.4%)	2.5%	(-14.4%, 19.4%)	3.3%	(-13.0%, 19.5%)
1	0.7%	(-16.9%, 18.4%)	2.8%	(-12.6%, 18.1%)	3.2%	(-12.1%, 18.4%)	-0.3%	(-19.7%, 19.1%)	0.4%	(-11.7%, 12.5%)	0.5%	(-16.0%, 17.1%)	2.3%	(-14.3%, 18.9%)
2	1.7%	(-16%, 19.3%)	4%	(-11.5%, 19.5%)	4.1%	(-11.1%, 19.3%)	0.4%	(-20.1%, 20.8%)	1.6%	(-11.1%, 14.4%)	1.9%	(-14.7%, 18.4%)	4.2%	(-12.0%, 20.3%)
3	0.4%	(-18.7%, 19.4%)	3%	(-13.9%, 19.8%)	3.0%	(-13.7%, 19.6%)	0.7%	(-20.4%, 21.8%)	1.3%	(-13.2%, 15.7%)	0.4%	(-17.3%, 18.0%)	2.9%	(-14.4%, 20.3%)
4	-0.2%	(-19%, 18.6%)	2.6%	(-14.1%, 19.3%)	2.6%	(-13.9%, 19.1%)	0.8%	(-19.5%, 21.1%)	0.9%	(-13.4%, 15.2%)	-0.2%	(-17.4%, 17.0%)	2.6%	(-14.4%, 19.5%)
5	-1.8%	(-22.6%, 18.9%)	1.1%	(-17.4%, 19.6%)	0.8%	(-17.5%, 19.2%)	0.1%	(-21.8%, 21.9%)	-0.3%	(-16.6%, 15.9%)	-2.5%	(-21.2%, 16.1%)	1.0%	(-17.7%, 19.7%)
6	-4.3%	(-26%, 17.5%)	-0.9%	(-20.4%, 18.6%)	-1.5%	(-20.9%, 18.0%)	-0.7%	(-22.7%, 21.3%)	-2.2%	(-19.2%, 14.9%)	-4.6%	(-23.8%, 14.6%)	-1.3%	(-20.7%, 18.2%)
7	-4.5%	(-27%, 18%)	-1.2%	(-21.5%, 19.1%)	-1.9%	(-22.1%, 18.3%)	-0.8%	(-23.3%, 21.7%)	-2.6%	(-20.8%, 15.5%)	-5.5%	(-25.6%, 14.6%)	-1.4%	(-21.7%, 18.9%)
8	-7.7%	(-31.5%, 16%)	-3.9%	(-25.2%, 17.4%)	-4.8%	(-26.1%, 16.4%)	-2.5%	(-25.6%, 20.7%)	-5.1%	(-24.4%, 14.1%)	-8.7%	(-29.5%, 12.1%)	-4.3%	(-25.6%, 17.0%)
9	-9.3%	(-34%, 15.3%)	-5.6%	(-27.7%, 16.6%)	-6.6%	(-28.9%, 15.7%)	-2.4%	(-26.0%, 21.3%)	-6.3%	(-26.5%, 14.0%)	-9.5%	(-31.2%, 12.2%)	-5.8%	(-28.0%, 16.4%)
10	-11.8%	(-36.9%, 13.2%)	-8.3%	(-31.1%, 14.4%)	-9.2%	(-32.0%, 13.6%)	-4.0%	(-28.5%, 20.6%)	-8.8%	(-30.0%, 12.4%)	-12.1%	(-34.2%, 9.9%)	-8.6%	(-31.6%, 14.3%)
11	-14.9%	(-41.4%, 11.6%)	-11.1%	(-35.2%, 12.9%)	-12.4%	(-36.6%, 11.7%)	-5.6%	(-31.4%, 20.1%)	-11.5%	(-34.5%, 11.5%)	-13.8%	(-37.4%, 9.8%)	-11.4%	(-36.0%, 13.2%)
12	-19.6%	(-47.8%, 8.6%)	-16.4%	(-42.8%, 10.0%)	-17.3%	(-43.5%, 9.0%)	-9.3%	(-39.0%, 20.3%)	-15.9%	(-42.0%, 10.2%)	-16.9%	(-42.0%, 8.3%)	-15.9%	(-43.6%, 11.7%)
13	-17.6%	(-45.4%, 10.2%)	-13.6%	(-38.2%, 11.0%)	-14.3%	(-38.9%, 10.4%)	-5.5%	(-31.6%, 20.7%)	-12.1%	(-36.4%, 12.2%)	-13.7%	(-38.8%, 11.4%)	-13.4%	(-39.1%, 12.3%)
14	-24.4%	(-57.9%, 9.1%)	-20.6%	(-50.9%, 9.7%)	-21.1%	(-51.2%, 9.1%)	-12.2%	(-46.4%, 22.1%)	-18.2%	(-48.2%, 11.8%)	-19.9%	(-50.8%, 11.1%)	-19.6%	(-52.2%, 13.0%)
15	-26.0%	(-59.2%, 7.2%)	-22.8%	(-55.0%, 9.4%)	-23.2%	(-55.1%, 8.7%)	-11.0%	(-48.0%, 26.0%)	-19.2%	(-49.5%, 11.1%)	-16.8%	(-49.1%, 15.5%)	-18.2%	(-53.1%, 16.7%)
16	-36.3%	(-82.5%, 9.9%)	-33.6%	(-78.8%, 11.6%)	-34.4%	(-78.2%, 9.4%)	-28.6%	(-82.4%, 25.1%)	-28.9%	(-73.6%, 15.9%)	-30.9%	(-75.6%, 13.8%)	-31.3%	(-79.1%, 16.5%)
17	-28.3%	(-65.8%, 9.2%)	-25.2%	(-59.9%, 9.5%)	-24.5%	(-59.4%, 10.4%)	-14.3%	(-47.2%, 18.5%)	-20.9%	(-50.8%, 8.9%)	-15.8%	(-51.5%, 19.9%)	-21.7%	(-58.4%, 15.1%)
18	-26.6%	(-83.1%, 29.9%)	-19.6%	(-71.8%, 32.6%)	-21.8%	(-74.3%, 30.7%)	-1.4%	(-42.8%, 40.1%)	-16.4%	(-61.3%, 28.5%)	-10.2%	(-59.0%, 38.6%)	-19.0%	(-69.5%, 31.4%)

© 2019 Grieve R et al. *JAMA Network Open*.

19	-25.4%	(-51.3%, 0.4%)	-25.8%	(-51.9%, 0.3%)	-23.9%	(-50.1%, 2.3%)	0.7%	(-24.2%, 25.7%)	-18.3%	(-36.9%, 0.4%)	-5.8%	(-33.9%, 22.2%)	-18.6%	(-47.8%, 10.6%)
**Treatment (outcome) equation**	**Probit (Probit)**		**Logit (Probit)**		**Logit (Logit)**		**Probit (Probit)**		**Probit (Probit)**		**Probit (Probit)**		**Probit (Probit)**	
**NEWS, SOFA & ICNARC scores**	**Yes**		**Yes**		**Yes**		**No**		**Yes**		**Yes**		**Yes**	
**NEWS, SOFA & ICNARC scores squared**	**Yes**		**Yes**		**Yes**		**No**		**No**		**No**		**Yes**	
**Interactions between NEWS, SOFA & ICNARC scores**	**No**		**No**		**No**		**No**		**No**		**Yes**		**Yes**	

© 2019 Grieve R et al. *JAMA Network Open*.

eTable 11: Sensitivity analysis of conditional average treatment effects on 90-day mortality, overall, and by subgroup.

	Baseline PeT Model 1		Model 2		Model 3		Model 4		Model 5		Model 6		Model 7	
	Incremental effect***	95% CI	Incremental effect***	95% CI	Incremental effect***	95% CI	Incremental effect***	95% CI	Incremental effect***	95% CI	Incremental effect***	95% CI	Incremental effect***	95% CI
**Overall average**	-4.7%	(-28.5%, 19.2%)	-2.5%	(-23.7%, 18.7%)	-2.5%	(-22.8%, 17.7%)	0.9%	(-22.0%, 23.8%)	-3.2%	(-22.9%, 16.6%)	-6%	(-27.1%, 15.1%)	-3.3%	(-24.9%, 18.4%)
**Type of care:**														
General ward	-0.5%	(-24.4%, 23.5%)	2.6%	(-19.0%, 24.3%)	2.8%	(-18.1%, 23.8%)	3.3%	(-19.9%, 26.5%)	1.7%	(-18.1%, 21.5%)	-0.8%	(-22.4%, 20.8%)	1.8%	(-19.9%, 23.4%)
ICU	-11.6%	(-36.9%, 13.6%)	-10.7%	(-33.2%, 11.8%)	-11.1%	(-32.3%, 10.2%)	-2.9%	(-28.4%, 22.5%)	-10.9%	(-32.7%, 10.9%)	-14.5%	(-36.7%, 7.6%)	-11.3%	(-35.2%, 12.6%)
**Age category**														
18-23	9.3%	(-7.9%, 26.5%)	11.1%	(-4.7%, 26.9%)	12.4%	(-2.8%, 27.5%)	12.2%	(-4.8%, 29.2%)	9.9%	(-4.9%, 24.7%)	7.3%	(-8.4%, 23.1%)	10.0%	(-6.4%, 26.3%)
24-29	8.8%	(-9.3%, 26.9%)	10.4%	(-6.0%, 26.8%)	11.8%	(-3.9%, 27.5%)	11.7%	(-5.5%, 29.0%)	9.5%	(-5.8%, 24.7%)	6.6%	(-9.6%, 22.9%)	9.4%	(-7.4%, 26.2%)
30-35	6.8%	(-11.1%, 24.6%)	8.2%	(-7.7%, 24.2%)	9.4%	(-5.6%, 24.4%)	10.2%	(-6.8%, 27.2%)	7.0%	(-7.7%, 21.7%)	4.7%	(-11.0%, 20.3%)	7.3%	(-9.0%, 23.7%)
36-41	3.4%	(-16.0%, 22.8%)	4.5%	(-13.0%, 22.1%)	5.8%	(-10.7%, 22.3%)	7.9%	(-10.8%, 26.7%)	3.9%	(-12.4%, 20.3%)	1.5%	(-15.6%, 18.5%)	3.6%	(-14.5%, 21.6%)
42-47	1.9%	(-17.8%, 21.6%)	3.2%	(-14.6%, 21.1%)	4.3%	(-12.5%, 21.1%)	6.2%	(-12.7%, 25.1%)	2.5%	(-13.9%, 19.0%)	-0.1%	(-17.8%, 17.7%)	2.7%	(-15.6%, 20.9%)
48-53	-0.1%	(-20.6%, 20.4%)	0.6%	(-18.1%, 19.3%)	1.3%	(-16.2%, 18.9%)	4.0%	(-16.0%, 24.1%)	0.3%	(-17.2%, 17.8%)	-2%	(-20.3%, 16.4%)	-0.2%	(-19.2%, 18.9%)
54-59	-3.1%	(-26.4%, 20.2%)	-2.1%	(-23.1%, 18.9%)	-1.9%	(-21.8%, 18.0%)	2.0%	(-20.5%, 24.6%)	-2.5%	(-22.1%, 17.1%)	-5.4%	(-25.9%, 15.1%)	-2.8%	(-24.3%, 18.7%)
60-65	-5.7%	(-29.3%, 18.0%)	-4.0%	(-25.3%, 17.4%)	-4.1%	(-24.4%, 16.3%)	0.2%	(-22.9%, 23.2%)	-4.4%	(-24.4%, 15.6%)	-7.1%	(-28.1%, 13.8%)	-4.8%	(-26.6%, 17.1%)
66-71	-7.2%	(-32.3%, 17.9%)	-5.2%	(-27.6%, 17.3%)	-5.5%	(-27.0%, 16.1%)	-1.4%	(-25.4%, 22.7%)	-5.8%	(-26.7%, 15.2%)	-8.7%	(-30.9%, 13.5%)	-6.0%	(-28.9%, 16.9%)
72-77	-8.6%	(-34.5%, 17.4%)	-6.4%	(-29.5%, 16.8%)	-7.0%	(-29.2%, 15.3%)	-2.2%	(-27.3%, 23.0%)	-6.8%	(-28.4%, 14.9%)	-9.6%	(-32.5%, 13.2%)	-7.2%	(-30.9%, 16.4%)
78-83	-9.4%	(-36.5%, 17.6%)	-6.4%	(-30.3%, 17.6%)	-7.1%	(-30.3%, 16.1%)	-2.6%	(-28.3%, 23.0%)	-7.2%	(-29.3%, 15.0%)	-10.3%	(-34.3%, 13.8%)	-7.2%	(-31.5%, 17.1%)
84-89	-8.3%	(-36.3%, 19.8%)	-4.4%	(-29.1%, 20.4%)	-5.0%	(-29.1%, 19.2%)	-1.8%	(-28.3%, 24.7%)	-5.5%	(-28.7%, 17.6%)	-8.6%	(-33.8%, 16.6%)	-5.0%	(-30.3%, 20.4%)
90-95	-6.1%	(-36.5%, 24.4%)	-1.2%	(-28.1%, 25.8%)	-1.4%	(-28.0%, 25.3%)	-0.5%	(-29.2%, 28.2%)	-2.7%	(-27.7%, 22.2%)	-5.8%	(-33.4%, 21.8%)	-1.6%	(-28.8%, 25.6%)

© 2019 Grieve R et al. *JAMA Network Open*.

96-101	-0.7%	(-33.7%, 32.3%)	4.3%	(-24.2%, 32.7%)	4.4%	(-24.0%, 32.9%)	3.3%	(-26.0%, 32.6%)	1.5%	(-25.7%, 28.7%)	-1.8%	(-32.1%, 28.4%)	3.4%	(-25.8%, 32.7%)
**NEWS risk category**														
**0**	2.9%	(-21.7%, 27.4%)	4.4%	(-17.0%, 25.8%)	4.9%	(-15.7%, 25.5%)	1.6%	(-20.1%, 23.3%)	1.4%	(-16.0%, 18.7%)	1.3%	(-20.0%, 22.6%)	3.2%	(-18.8%, 25.2%)
**1**	-0.1%	(-22.8%, 22.6%)	2.1%	(-18.0%, 22.1%)	2.3%	(-17.0%, 21.7%)	2.1%	(-19.9%, 24.0%)	0.4%	(-17.6%, 18.3%)	-1.1%	(-21.7%, 19.4%)	1.5%	(-19.1%, 22.0%)
**2**	-3.3%	(-26.8%, 20.2%)	-1.0%	(-22.0%, 20.1%)	-0.9%	(-21.1%, 19.3%)	1.6%	(-21.1%, 24.2%)	-1.7%	(-21.4%, 18.0%)	-4.5%	(-25.5%, 16.5%)	-1.7%	(-22.9%, 19.5%)
**3**	-8.8%	(-34.0%, 16.4%)	-6.6%	(-29.3%, 16.0%)	-6.9%	(-28.5%, 14.7%)	-0.2%	(-24.4%, 23.9%)	-6.5%	(-28%, 15.0%)	-10.3%	(-32.3%, 11.7%)	-7.5%	(-30.8%, 15.8%)
**NEWS score**														
0	3.2%	(-21.9%, 28.2%)	5.0%	(-16.8%, 26.8%)	5.5%	(-15.6%, 26.5%)	1.4%	(-20.2%, 23.1%)	1.5%	(-15.8%, 18.9%)	1.4%	(-20.1%, 22.9%)	3.8%	(-18.5%, 26.1%)
1	1.1%	(-22.0%, 24.3%)	3.0%	(-17.3%, 23.3%)	3.5%	(-16.2%, 23.2%)	0.6%	(-20.3%, 21.5%)	-0.3%	(-16.7%, 16.1%)	-1%	(-21.2%, 19.2%)	2.2%	(-18.7%, 23.1%)
2	1.3%	(-21.4%, 24.0%)	3.3%	(-16.7%, 23.2%)	3.6%	(-15.6%, 22.8%)	1.9%	(-19.7%, 23.6%)	0.6%	(-16.3%, 17.6%)	-0.1%	(-20.6%, 20.3%)	2.7%	(-17.9%, 23.4%)
3	0.6%	(-22.7%, 23.9%)	2.8%	(-17.9%, 23.5%)	3.1%	(-16.9%, 23.1%)	2.5%	(-19.9%, 24.9%)	0.9%	(-17.9%, 19.7%)	-0.5%	(-21.6%, 20.7%)	2.1%	(-19.1%, 23.2%)
4	-0.7%	(-22.8%, 21.4%)	1.9%	(-17.9%, 21.7%)	2.1%	(-17.0%, 21.2%)	2.1%	(-19.4%, 23.6%)	0.4%	(-17.8%, 18.6%)	-1.5%	(-21.7%, 18.7%)	1.2%	(-18.8%, 21.1%)
5	-1.9%	(-25.3%, 21.5%)	0.4%	(-20.5%, 21.4%)	0.6%	(-19.5%, 20.7%)	2.2%	(-20.6%, 25.1%)	-0.5%	(-20.1%, 19.1%)	-3.4%	(-24.6%, 17.7%)	-0.3%	(-21.6%, 20.9%)
6	-4.6%	(-28.3%, 19.2%)	-2.1%	(-23.3%, 19.1%)	-2.1%	(-22.5%, 18.3%)	1.2%	(-21.5%, 24.0%)	-2.5%	(-22.5%, 17.5%)	-5.5%	(-26.6%, 15.6%)	-2.9%	(-24.3%, 18.5%)
7	-4.7%	(-28.9%, 19.6%)	-2.3%	(-24.2%, 19.6%)	-2.5%	(-23.5%, 18.5%)	1.7%	(-21.4%, 24.8%)	-2.7%	(-23.4%, 18.0%)	-6.3%	(-28.0%, 15.4%)	-3.2%	(-25.3%, 19.0%)
8	-7.9%	(-32.4%, 16.7%)	-5.2%	(-27.3%, 16.9%)	-5.5%	(-26.6%, 15.6%)	0.2%	(-23.3%, 23.6%)	-5.3%	(-26.3%, 15.8%)	-9.6%	(-31.1%, 12.0%)	-6.2%	(-28.7%, 16.3%)
9	-8.7%	(-33.9%, 16.6%)	-6.5%	(-29.1%, 16.1%)	-6.7%	(-28.4%, 14.9%)	0.4%	(-23.3%, 24.2%)	-6.0%	(-27.5%, 15.5%)	-10%	(-32.1%, 12.1%)	-7.3%	(-30.4%, 15.8%)
10	-10.9%	(-36.4%, 14.5%)	-9.2%	(-32.2%, 13.9%)	-9.5%	(-31.4%, 12.4%)	-1.3%	(-26.0%, 23.5%)	-8.7%	(-30.7%, 13.3%)	-12.8%	(-34.7%, 9.1%)	-10.2%	(-33.9%, 13.5%)
11	-13.6%	(-40.8%, 13.7%)	-11.5%	(-36.0%, 12.9%)	-12.0%	(-35.3%, 11.2%)	-2.2%	(-28.0%, 23.6%)	-10.7%	(-33.9%, 12.5%)	-14.4%	(-37.6%, 8.8%)	-12.3%	(-37.8%, 13.1%)
12	-16.3%	(-45.1%, 12.6%)	-15.2%	(-42.1%, 11.6%)	-15.5%	(-40.8%, 9.7%)	-4.2%	(-34.6%, 26.2%)	-13.7%	(-39.4%, 12.0%)	-16.5%	(-40.6%, 7.6%)	-15.3%	(-43.6%, 13.0%)

© 2019 Grieve R et al. *JAMA Network Open*.

13	-13.6%	(-42.2%, 14.9%)	-12.0%	(-37.2%, 13.1%)	-12.4%	(-36.2%, 11.4%)	-1.1%	(-27.3%, 25.0%)	-10.4%	(-34.0%, 13.2%)	-13.5%	(-37.2%, 10.3%)	-12.1%	(-38.9%, 14.6%)
14	-18.6%	(-52.3%, 15.2%)	-17.1%	(-47.4%, 13.2%)	-17.3%	(-46.0%, 11.5%)	-5.6%	(-40.9%, 29.6%)	-14.7%	(-43.5%, 14.1%)	-18.6%	(-46.8%, 9.7%)	-16.7%	(-49.5%, 16.1%)
15	-19.3%	(-51.4%, 12.8%)	-18.6%	(-49.8%, 12.6%)	-18.5%	(-46.8%, 9.7%)	-5.7%	(-44.5%, 33.1%)	-14.9%	(-43.5%, 13.7%)	-16.5%	(-44.9%, 11.9%)	-16.4%	(-50.3%, 17.5%)
16	-27.7%	(-73.2%, 17.8%)	-27.3%	(-71.9%, 17.3%)	-27.6%	(-69.4%, 14.1%)	-18.5%	(-73.5%, 36.6%)	-27.7%	(-69.2%, 13.8%)	-29.6%	(-69.3%, 10.0%)	-26.3%	(-74.7%, 22.1%)
17	-17.5%	(-56.1%, 21.2%)	-17.2%	(-52.2%, 17.9%)	-16.4%	(-50.9%, 18.1%)	-7.5%	(-41.3%, 26.3%)	-13.9%	(-42.8%, 15.1%)	-13.3%	(-45.3%, 18.8%)	-16.0%	(-52.4%, 20.4%)
18	-19.7%	(-68.6%, 29.3%)	-17.7%	(-63.9%, 28.4%)	-18.1%	(-61.8%, 25.5%)	2.3%	(-38.1%, 42.7%)	-16.7%	(-51.6%, 18.2%)	-17.7%	(-58.8%, 23.5%)	-11.0%	(-55.3%, 33.4%)
19	-18.4%	(-42.3%, 5.5%)	-19.1%	(-41.1%, 2.8%)	-18.1%	(-38.5%, 2.2%)	-2.6%	(-25.4%, 20.3%)	-16.4%	(-30.5%, -2.3%)	-11.4%	(-33.2%, 10.4%)	-15.7%	(-41.1%, 9.7%)
**Functional form for 1st treatment (outcome) equation**	**Probit (Probit)**		**Logit (Probit)**		**Logit (Logit)**		**Probit (Probit)**		**Probit (Probit)**		**Probit (Probit)**		**Probit (Probit)**	
**NEWS, SOFA & ICNARC scores**	**Yes**		**Yes**		**Yes**		**No**		**Yes**		**Yes**		**Yes**	
**NEWS, SOFA & ICNARC scores squared**	**Yes**		**Yes**		**Yes**		**No**		**No**		**No**		**Yes**	
**Interactions between NEWS, SOFA & ICNARC scores**	**No**		**No**		**No**		**No**		**No**		**Yes**		**Yes**	

© 2019 Grieve R et al. *JAMA Network Open*.

eTable 12: 28- and 90-day Mortality following ICU versus General Ward care for the matched sample excluding patients transferred after 24 hours.

	Sample size*	ICU, Deaths N** (*%)	General Ward, Deaths N** (%)	Risk difference***	
		N(%)	N(%)	Mean	[95% CI]**
28 day, n (%)	7,894	2092.7 (26.5%)	1799.3 (22.8%)	3.7%	(-95.0% to 52.8%)
90 day, n (%)	7,893	2757.2 (34.9%)	2229.6 (28.2%)	6.7%	(-97.8% to 43.8%)

*For each method, the maximum sample size was 9,192. Observations were excluded if there is not mass at any value (rounded to 0.01) of the propensity score for both levels of exposure as recommended by Basu (2015). **The number of predicted deaths is rounded to the nearest whole number. *** Difference in percentage of deaths from the PeT Instrumental variable estimate. *** Normal based CI with SE calculated with the non-parametric bootstrap allowing for clustering by hospital. *** difference in percentage of deaths from the PeT Instrumental variable estimate ** Normal based CI with SE calculated with the non-parametric bootstrap allowing for clustering by hospital

ICU, intensive care unit; CI, confidence interval; IV, Instrumental Variable; PeT, Person-centred treatment effect;

© 2019 Grieve R et al. *JAMA Network Open*.

eTable 13: 28- and 90-day day Mortality following ICU versus General Ward care for the matched sample excluding patients recommended for ICU (level 3)

	Sample size*	ICU, Deaths N** (*%)	General Ward, Deaths N** (%)	Risk difference***	
		N(%)	N(%)	Mean	[95% CI]**
28 day, n (%)	8,271	1506.4 (18.2%)	2166.7 (26.2%)	-8.0%	(-31.8 to 15.8%)
90 day, n (%)	8,269	1938.9 (23.4%)	2707.2 (32.7%)	-9.3%	(-34.5 to 15.9%)

*For each method, the maximum sample size was 9,192. Observations were excluded if there is not mass at any value (rounded to 0.01) of the propensity score for both levels of exposure as recommended by Basu (2015). **The number of predicted deaths is rounded to the nearest whole number. *** Difference in percentage of deaths from the PeT Instrumental variable estimate. *** Normal based CI with SE calculated with the non-parametric bootstrap allowing for clustering by hospital. *** difference in percentage of deaths from the PeT Instrumental variable estimate ** Normal based CI with SE calculated with the non-parametric bootstrap allowing for clustering by hospital

ICU, intensive care unit; CI, confidence interval; IV, Instrumental Variable; PeT, Person-centred treatment effect;

© 2019 Grieve R et al. *JAMA Network Open*.

eFigure 1: Mean level of rescaled variables according to the level of the instrumental variable.

a) Continuous covariates

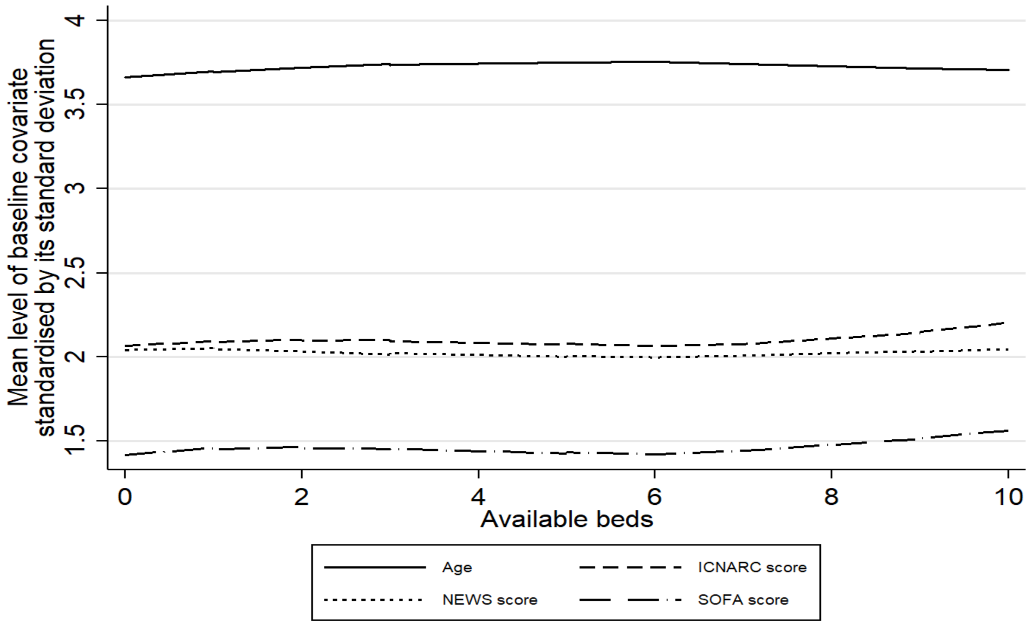

© 2019 Grieve R et al. *JAMA Network Open*.

b) Binary covariates

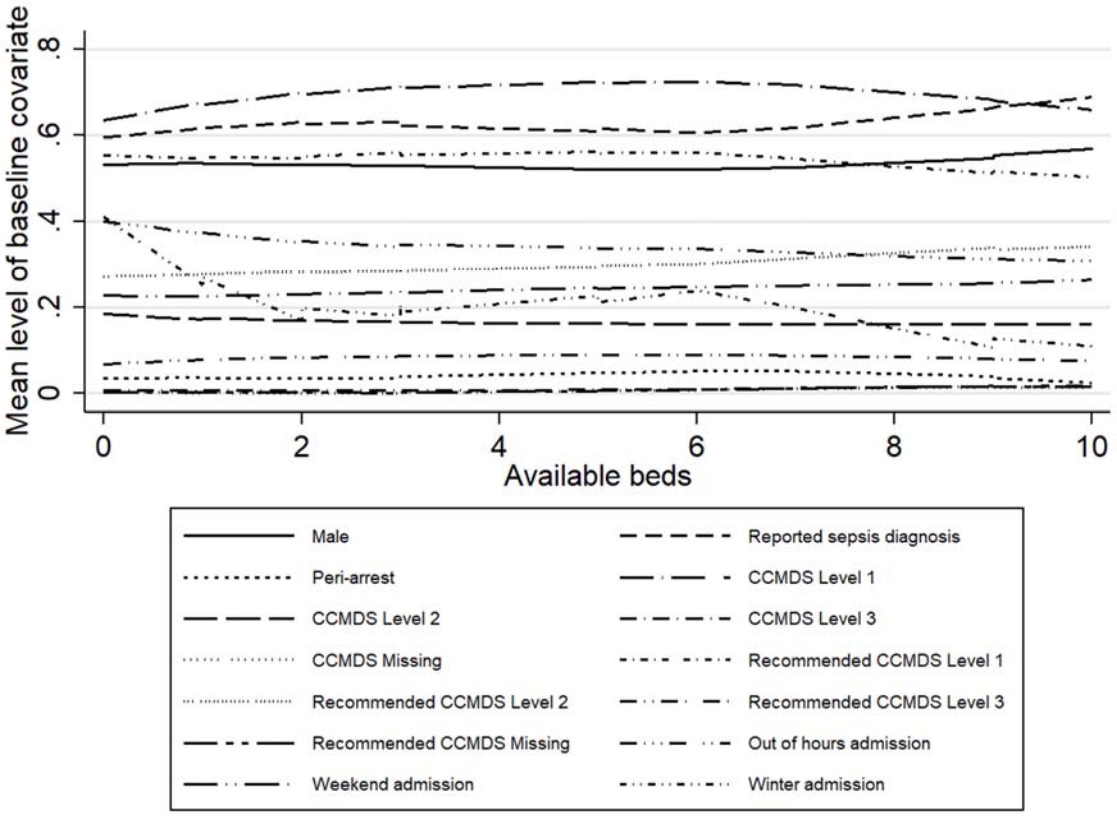

© 2019 Grieve R et al. *JAMA Network Open*.

eFigure 2: Illustration of plausible values for unobserved covariates influencing transfer decision.

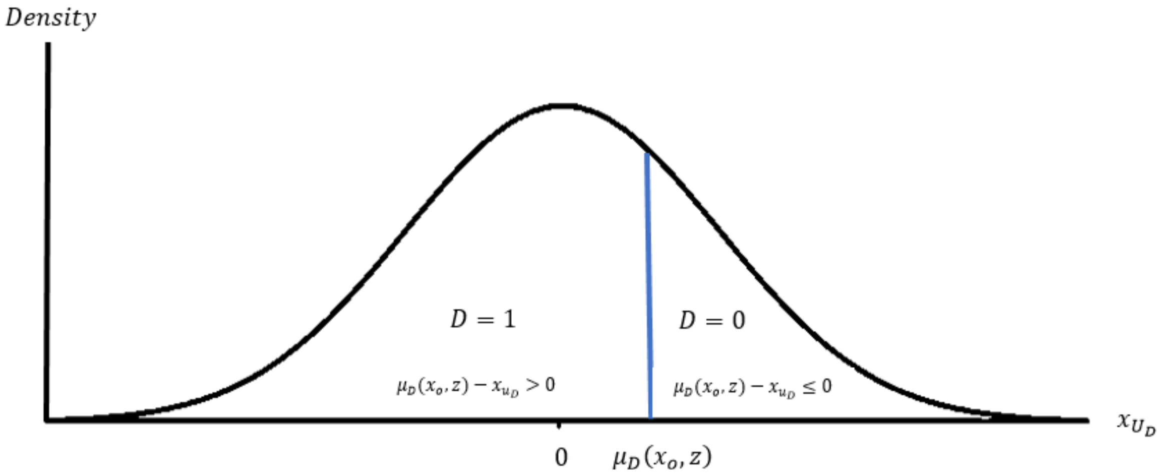

In the example above, an individual with particular values for their observed covariates (x_o) with *z* beds available at the time of their assessment for transfer, would be transferred to ICU if there were no unobserved factors which discourage transfer since the $\mu_D(x_o, z)$ is greater than 0 (see equation 2). If the unobserved risk factors that discourage transfer were non-zero, but smaller in magnitude than $\mu_D(x_o, z)$, then their effect would be insufficient to offset that of the factors that encourage transfer, and the individual would be transferred. The probability this occurs is represented by the area under the probability density curve to the left of the blue line. If instead, the discouraging factors exceeded this level, then the individual would no longer be transferred to ICU. The probability this occurs is represented by the area under the curve to the right of the blue line. Thus the observed choice as to whether the individual is transferred is informative about the level of the unobserved variables X_{UD}.

© 2019 Grieve R et al. *JAMA Network Open*.

eFigure 3: Estimated PeT effects of ICU transfer versus general ward care on 28-day mortality, overall, and by subgroup according to whether ICU transfer was recommended, and whether the patient was actually transferred.

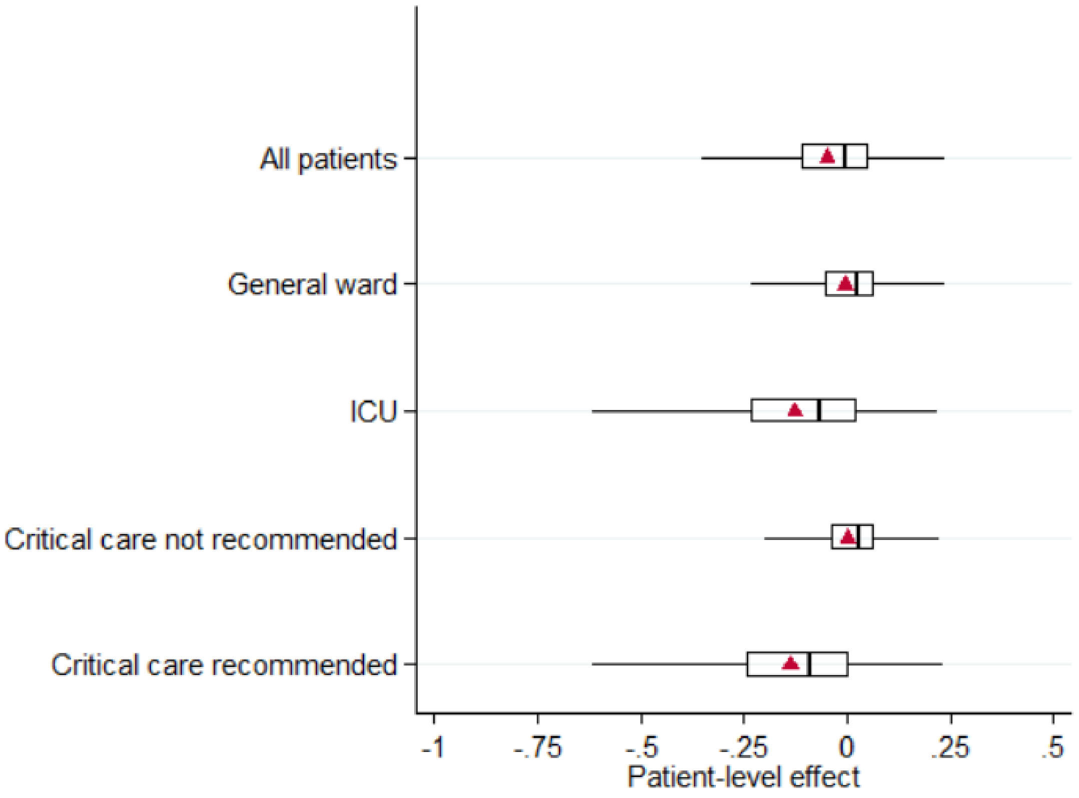

© 2019 Grieve R et al. *JAMA Network Open*.

eFigure 4: Bubble chart indicating average effect of being transferred to ICU by other subgroups.

Dark green indicates levels of absolute risk reductions > 10%, light green 0 to 10% risk reduction and red indicates increased absolute risk. Larger dot indicates more individuals in that subgroup.

(a) **ICNARC score by age category**

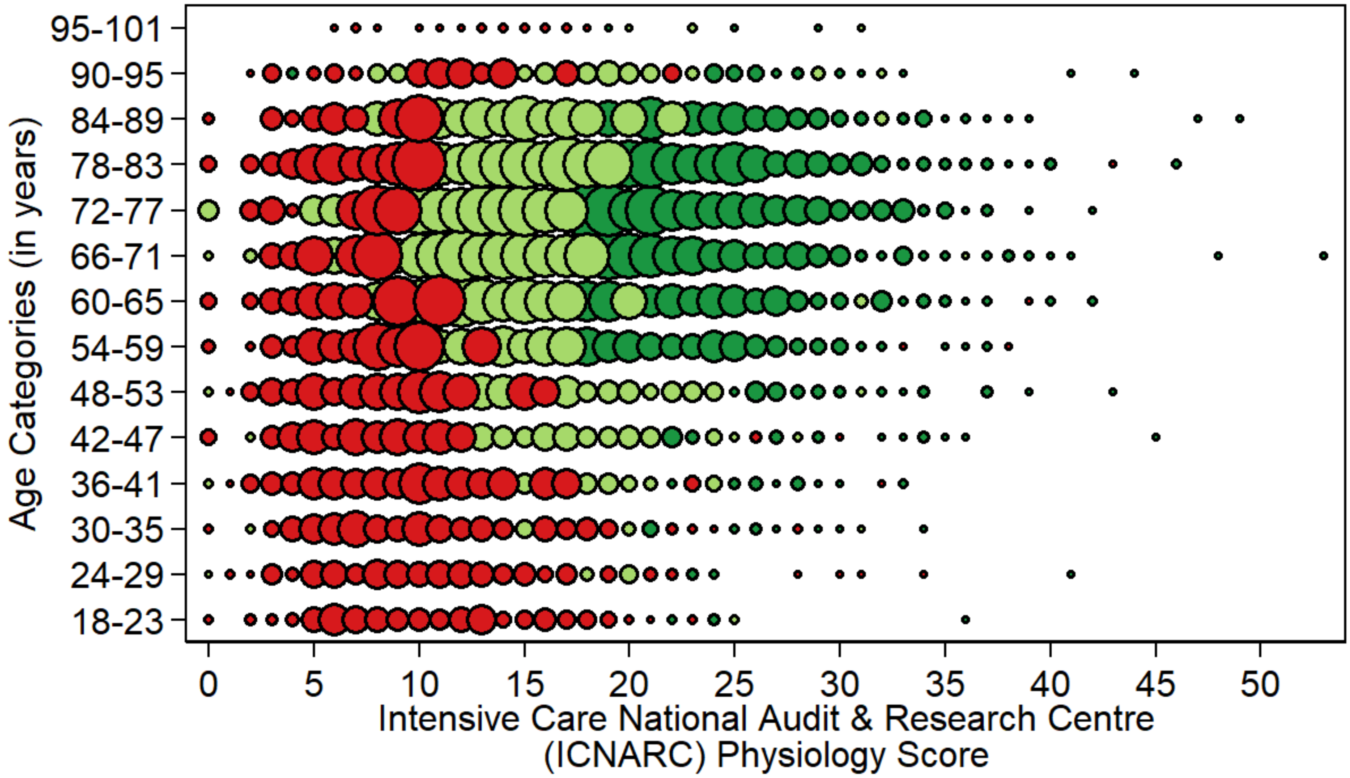

© 2019 Grieve R et al. *JAMA Network Open*.

(b) **SOFA score by age category**

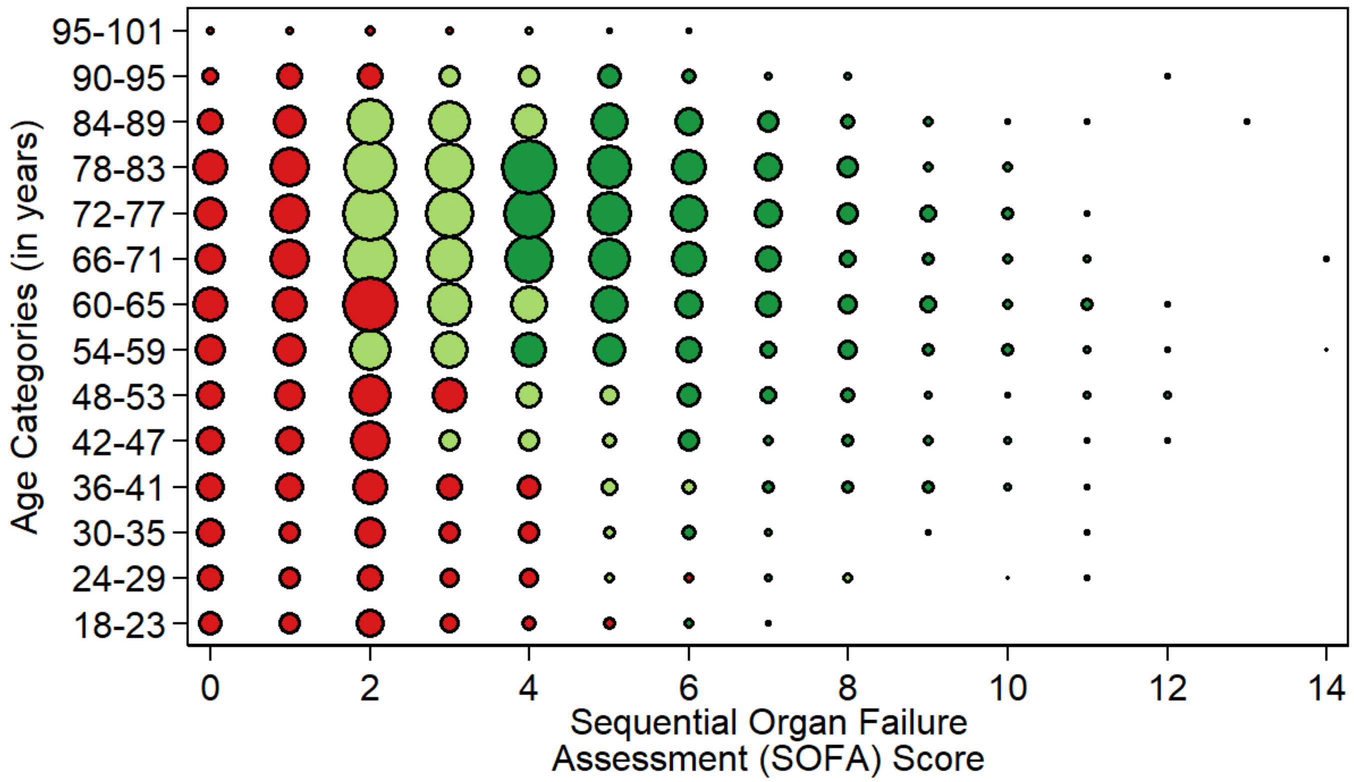

© 2019 Grieve R et al. *JAMA Network Open*.

(c) **NEWS score by ICNARC score**

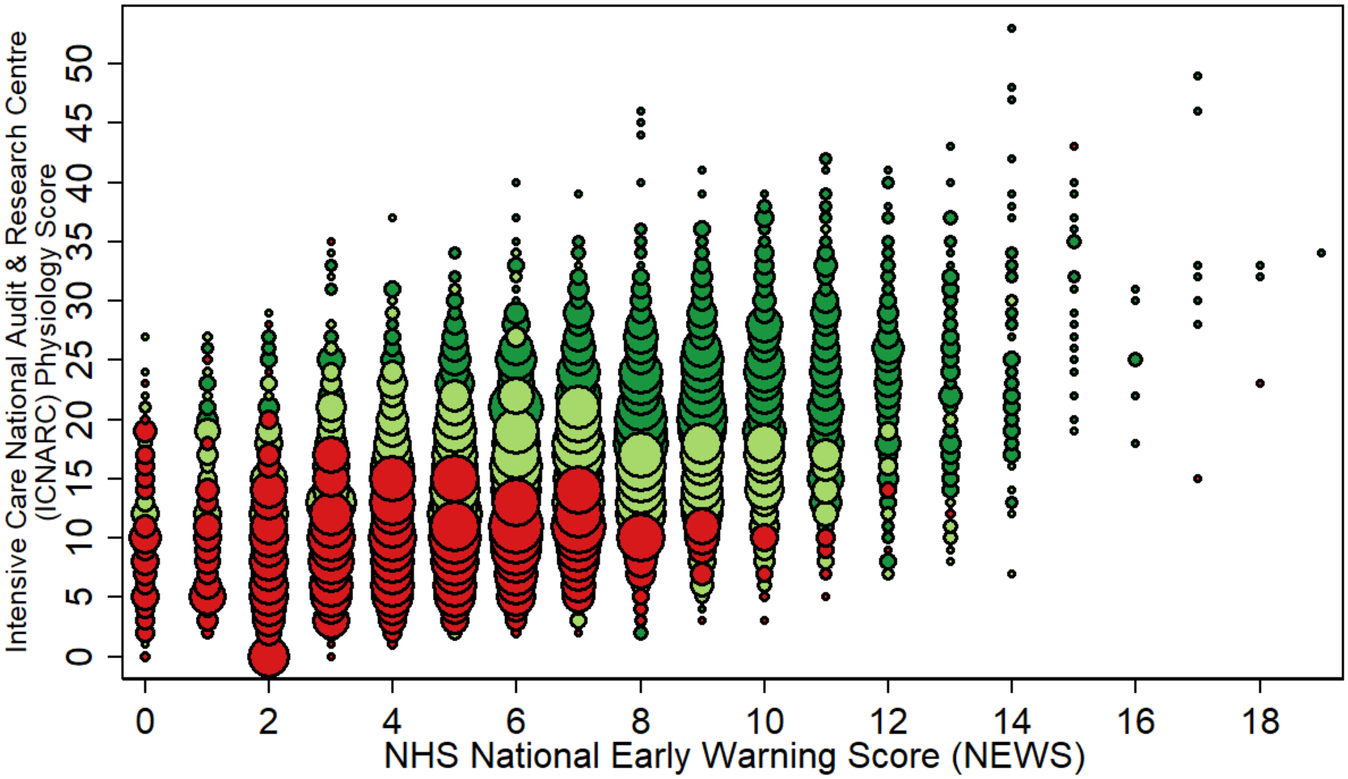

© 2019 Grieve R et al. *JAMA Network Open*.

d) ICNARC score by SOFA score

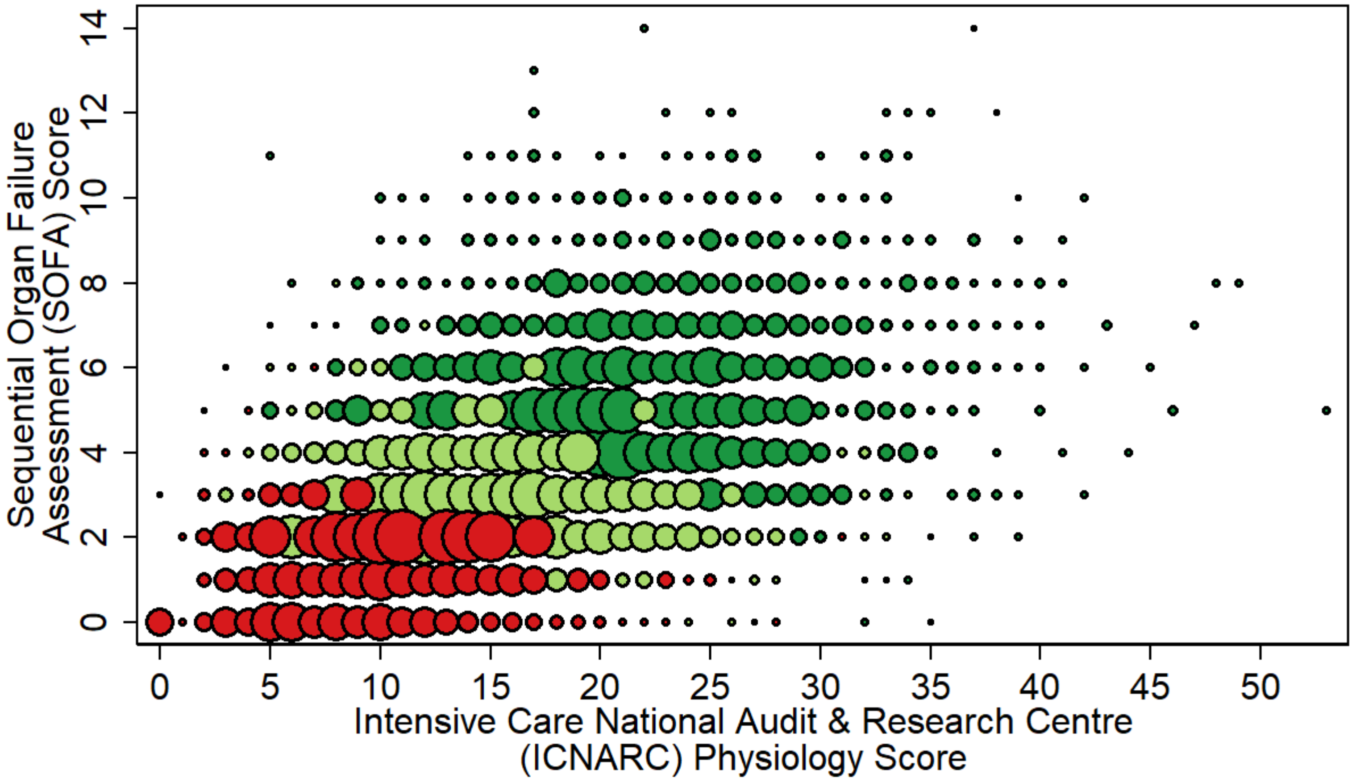

© 2019 Grieve R et al. *JAMA Network Open*.

e) **NEWS score by SOFA score**

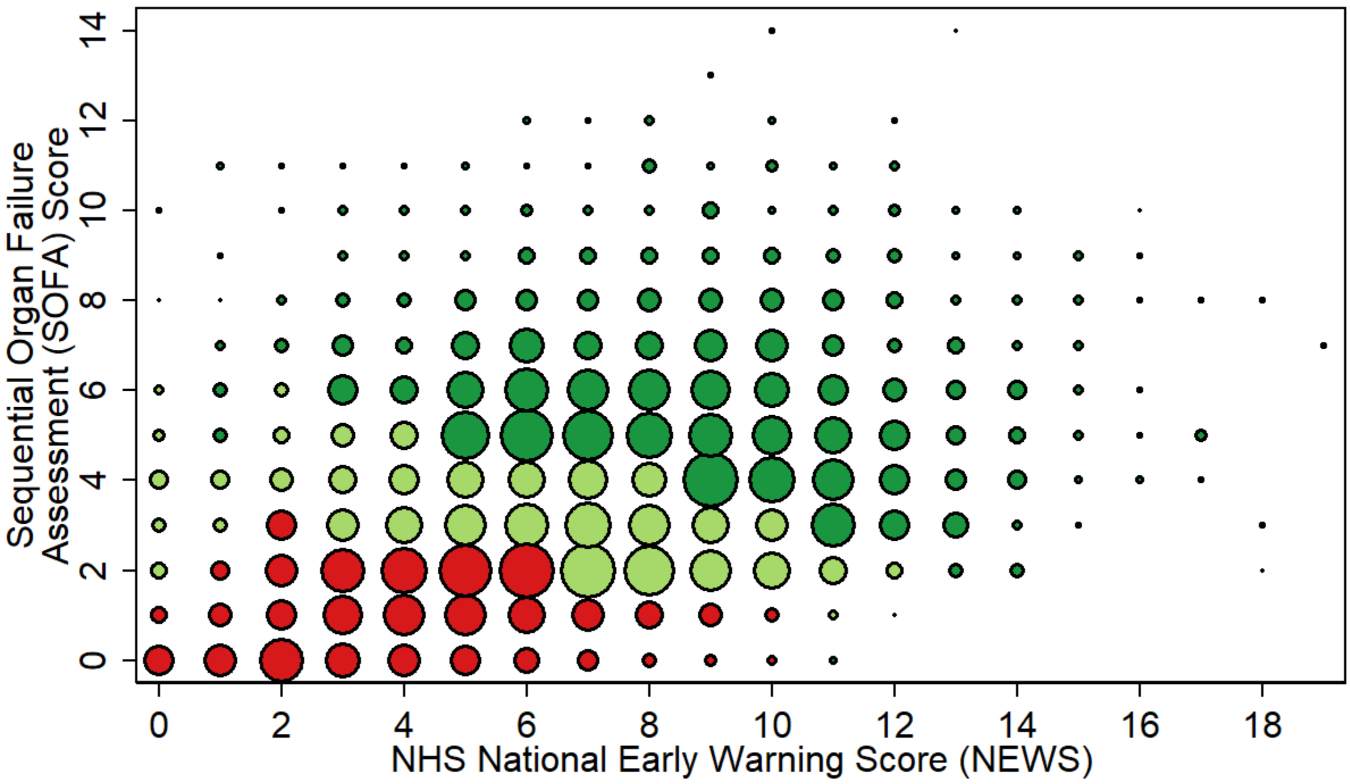

© 2019 Grieve R et al. *JAMA Network Open*.

eFigure 5: Estimated PeT effects of ICU transfer versus general ward care on 90-day mortality, according to strata, defined by age and physiology scores at assessment for ICU transfer.

Absolute risk reductions (95% CI).

A: 90 day mortality by age category

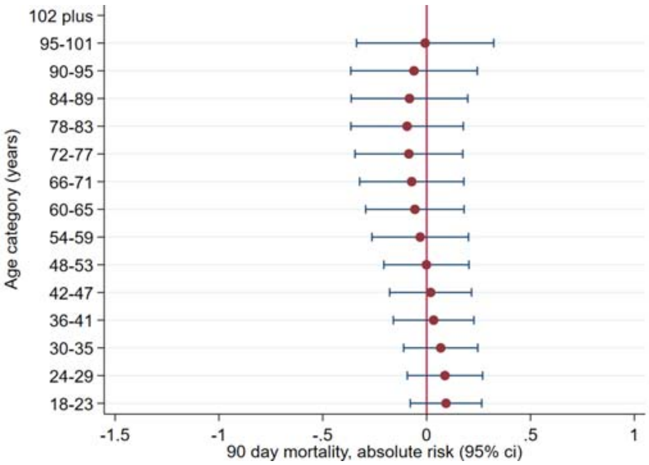

B: 90 day mortality by NEWS score

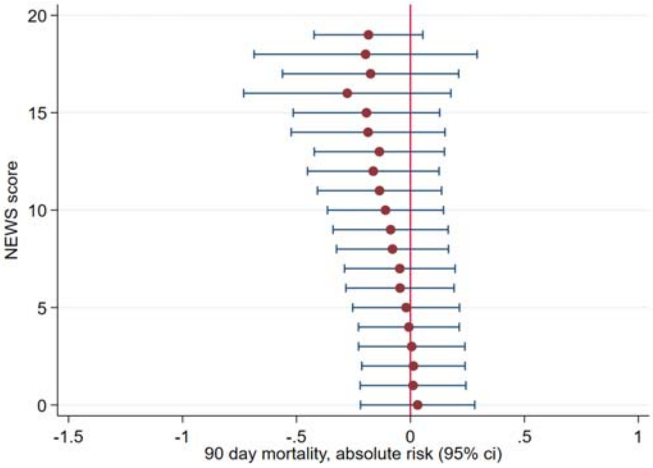

C: 90 day mortality by ICNARC score

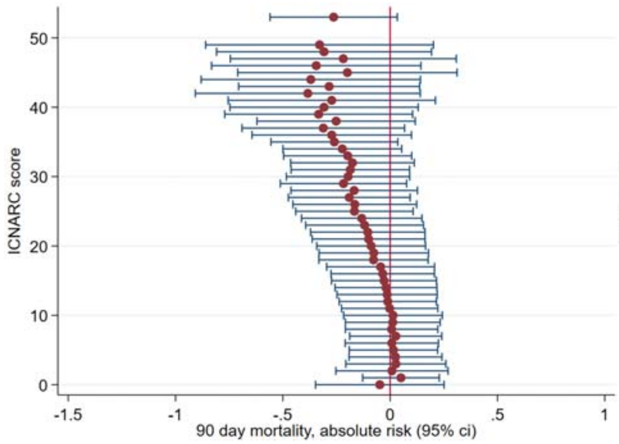

D: 90 day mortality by SOFA score

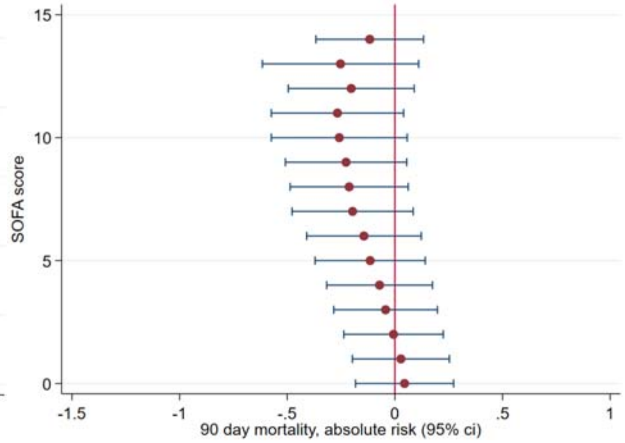

Note: Heterogeneous effects are estimated for each individual using the PeT method and then aggregated according to strata. The NHS National Early Warning Score (NEWS) ranges from 0 (least severe) to 20 (most severe). The Sequential Organ Failure Assessment (SOFA) ranges from 0 (least severe) to 14 (most severe), and the Intensive Care National Audit & Research Centre (ICNARC) physiology score ranges from 0 (least severe) to 100 (most severe).

© 2019 Grieve R et al. *JAMA Network Open*.

eFigure 6: Sensitivity analysis, excluding patients transferred to ICU more than 24 hours after admission. Bubble chart indicating estimated PeT effects of ICU transfer versus general ward care on 28-day mortality, by age category and NEWS score.

Dark green indicates levels of absolute risk reductions for 28-day mortality > 10%, light green 0 to 10% risk reduction and red indicates increased absolute risk. Larger dot indicates more individuals in that subgroup. Left of the dashed lines indicates 'low' risk, between the dashed lines 'medium' risk and to the right of the dashed lines, 'high' risk.

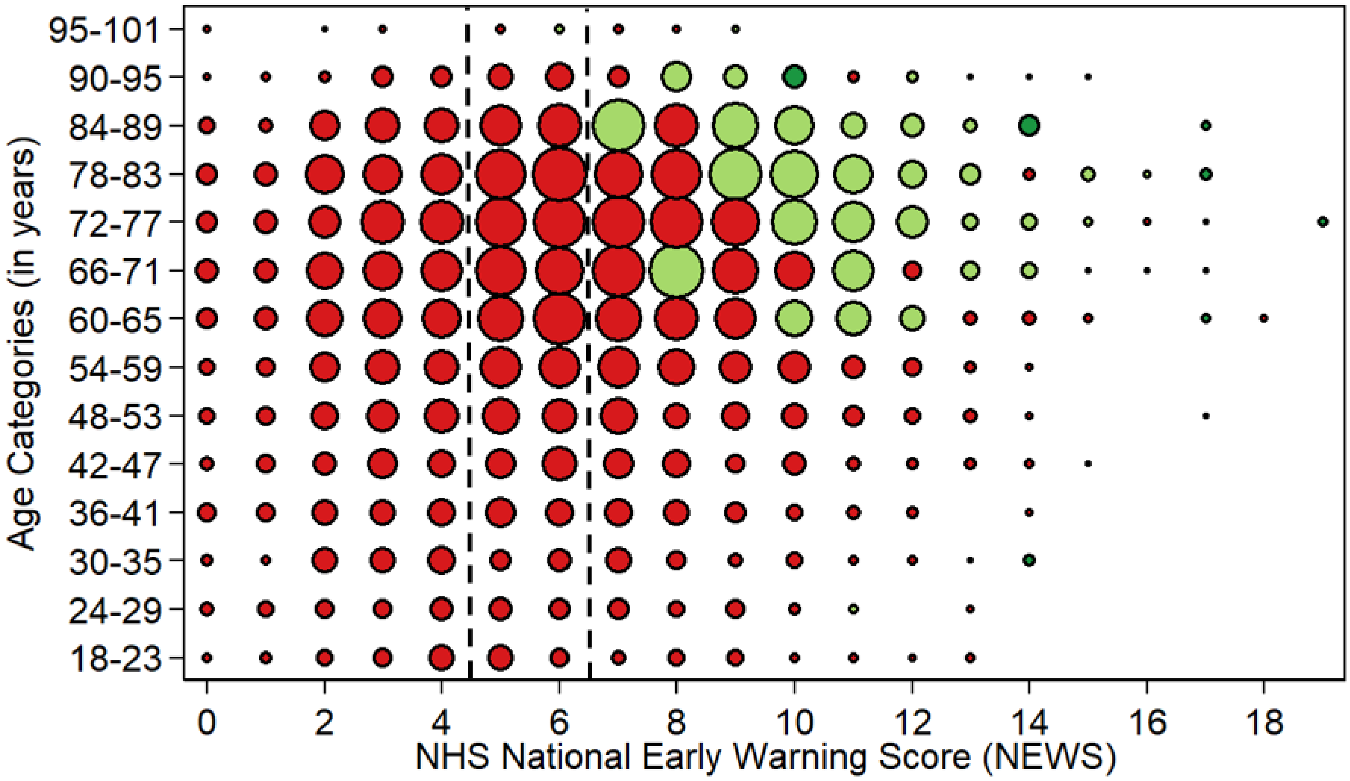

© 2019 Grieve R et al. *JAMA Network Open*.

eFigure 7: Sensitivity analysis, excluding patients recommended for level 3 (ICU) care post assessment. Bubble chart indicating estimated PeT effects of ICU transfer versus general ward care on 28-day mortality, by age category and NEWS score.

Dark green indicates levels of absolute risk reductions > 10%, light green 0 to 10% risk reduction and red indicates increased absolute risk. Larger dot indicates more individuals in that subgroup. Left of the dashed lines indicates ‘low’ risk, between the dashed lines ‘medium’ risk and to the right of the dashed lines, ‘high’ risk.

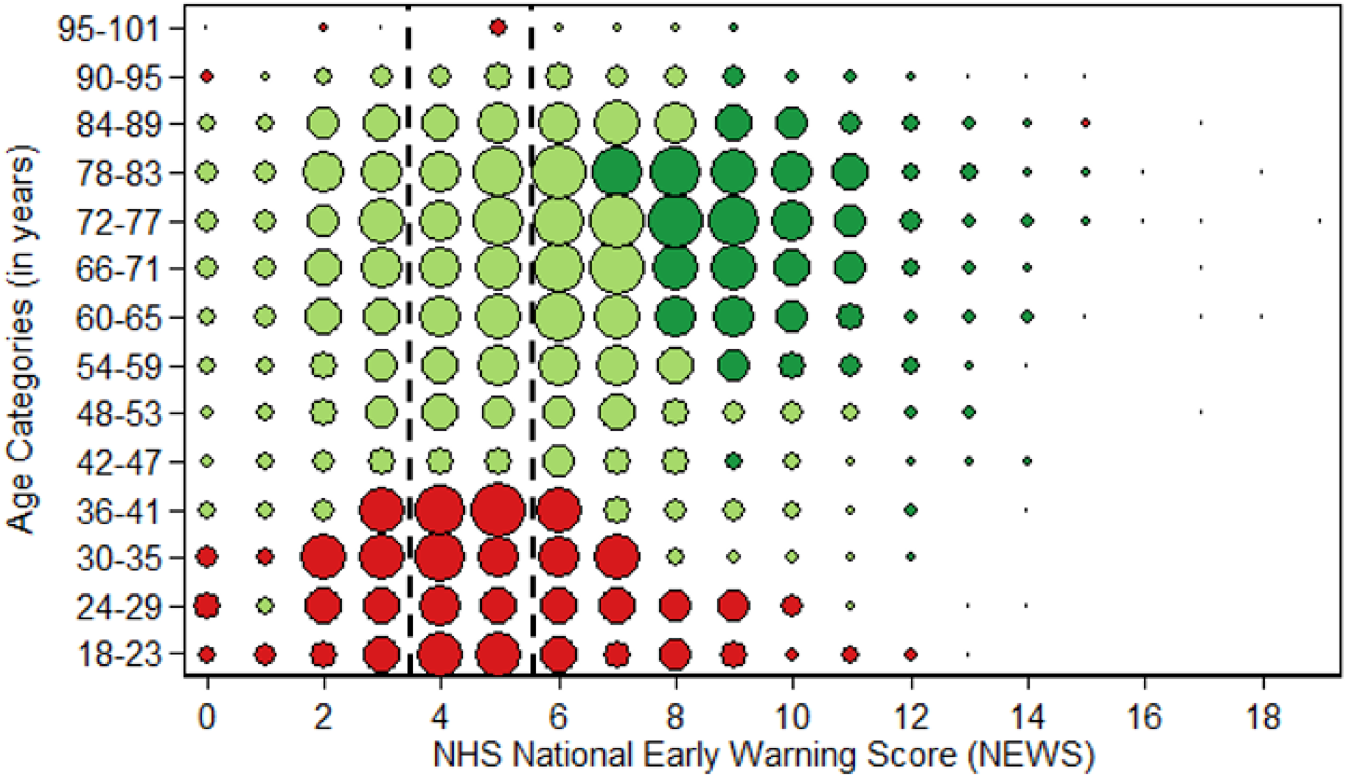

© 2019 Grieve R et al. *JAMA Network Open*.

eFigure 8: **Distribution of potential effects of transfer to ICU on 28- and 90-day mortality according to actual admission.**

(a) 28 day mortality

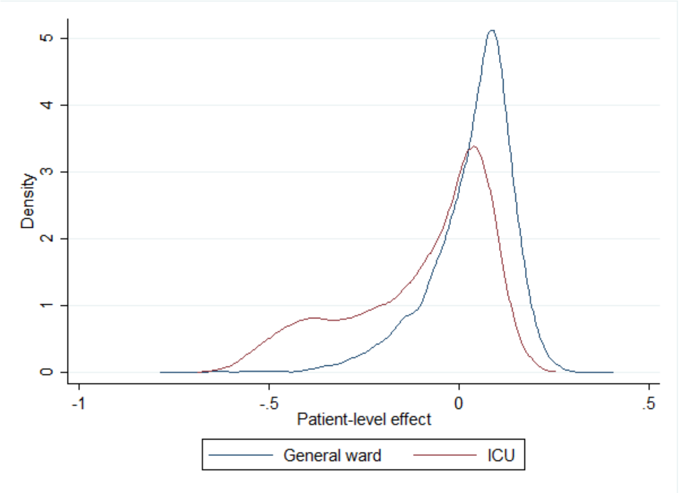

(b) 90 day mortality

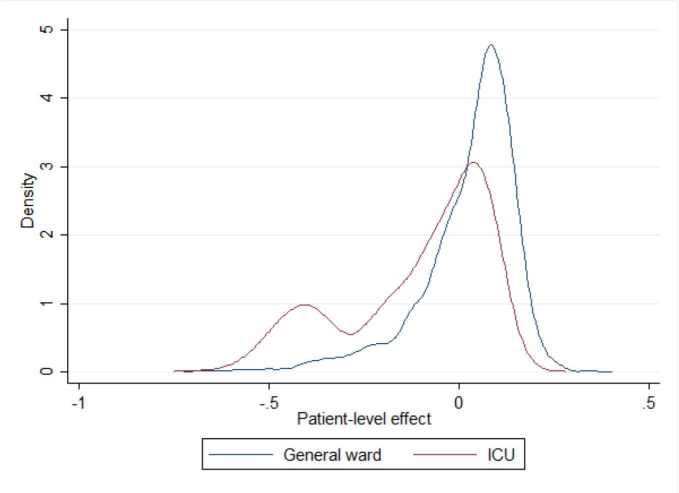

© 2019 Grieve R et al. *JAMA Network Open*.
